# Identification of an α-(1**→**6)-Mannosyltransferase Contributing To Biosynthesis of the Fungal-Type Galactomannan α-Core-Mannan Structure in Aspergillus fumigatus

**DOI:** 10.1128/msphere.00484-22

**Published:** 2022-11-29

**Authors:** Chihiro Kadooka, Daisuke Hira, Yutaka Tanaka, Ken Miyazawa, Masaaki Bise, Shogo Takatsuka, Takuji Oka

**Affiliations:** a Department of Biotechnology and Life Sciences, Sojo University, Kumamoto, Japan; b Division of Infection and Host Defense, Tohoku Medical and Pharmaceutical University, Sendai, Japan; c Department of Fungal Infection, National Institute of Infectious Diseases, Tokyo, Japan; University of Georgia

**Keywords:** galactomannan, cell wall, mannosyltransferase, glycosyltransferase, *Aspergillus fumigatus*, mannose, galactofuranose

## Abstract

Fungal-type galactomannan, a cell wall component of Aspergillus fumigatus, is composed of α-(1→2)-/α-(1→6)-linked mannan and β-(1→5)-/β-(1→6)-linked galactofuran side chains. Recently, CmsA and CmsB were identified as the α-(1→2)-mannosyltransferases involved in the biosynthesis of the α-core-mannan. However, the α-(1→6)-mannosyltransferase involved in the biosynthesis of the α-core-mannan has not been identified yet. In this study, we analyzed 9 putative α-(1→6)-mannosyltransferase gene disruption strains of A. fumigatus. The Δ*anpA* strain resulted in decreased mycelial elongation and reduced conidia formation. Proton nuclear magnetic resonance analysis revealed that the Δ*anpA* strain failed to produce the α-core-mannan of fungal-type galactomannan. We also found that recombinant AnpA exhibited much stronger α-(1→6)-mannosyltransferase activity toward α-(1→2)-mannobiose than α-(1→6)-mannobiose in vitro. Molecular simulations corroborated the fact that AnpA has a structure that can recognize the donor and acceptor substrates suitable for α-(1→6)-mannoside bond formation and that its catalytic activity would be specific for the elongation of the α-core-mannan structure *in vivo*. The identified AnpA is similar to Anp1p, which is involved in the elongation of the N-glycan outer chain in budding yeast, but the building sugar chain structure is different. The difference was attributed to the difference in substrate recognition of AnpA, which was clarified by simulations based on protein conformation. Thus, even proteins that seem to be functionally identical due to amino acid sequence similarity may be glycosyltransferase enzymes that make different glycans upon detailed analysis. This study describes an example of such a case.

**IMPORTANCE** Fungal-type galactomannan is a polysaccharide incorporated into the cell wall of filamentous fungi belonging to the subphylum Pezizomycotina. Biosynthetic enzymes of fungal-type galactomannan are potential targets for antifungal drugs and agrochemicals. In this study, we identified an α-(1→6)-mannosyltransferase responsible for the biosynthesis of the α-core-mannan of fungal-type galactomannan, which has not been known for a long time. The findings of this study shed light on processes that shape this cellular structure while identifying a key enzyme essential for the biosynthesis of fungal-type galactomannan.

## INTRODUCTION

Galactomannan (GM), composed of d-mannose (Man) and d-galactofuranose residues, is a component of the cell wall of filamentous fungi (1–4). Aspergillus fumigatus, the major pathogenic fungus causing invasive pulmonary aspergillosis, contains 2 types of GMs: fungal-type galactomannan (FTGM) and O-mannose-type galactomannan (OMGM) (5). FTGM has a linear α-mannan structure, called α-core-mannan, consisting of 9–10 α-(1→2)-mannotetraose units linked by α-(1→6)-bonds (6, 7). FTGM also contains galactofuran side chains comprising β-(1→5)-/β-(1→6)-galactofuranosyl chains linked to the α-core-mannan by β-(1→2), β-(1→3), and/or β-(1→6)-bonds (6, 7). FTGMs are thought to be biosynthesized in the Golgi apparatus, using glycosylphosphatidylinositol (GPI) anchors as carrier molecules, and transported to the cell membrane (8, 9), where they are cleaved and covalently bound to β-(1→3)-glucan in the cell wall (10), while a part of them is released into the extracellular medium. OMGM consists of β-(1→5)-galactofuranosyl chains bound to the nonreducing terminal side of an O-mannose-type glycan, where mannosyl chains are attached to a hydroxyl group of serine or threonine in the proteins (7, 11, 12). Like FTGM, the galactofuranosyl residues of OMGM are elongated by β-(1→6)-linked galactofuranose.

The β-(1→5)-galactofuranosyl chains observed in both FTGM and OMGM are biosynthesized by the enzymes GfsA and GfsC (5, 13, 14). Recently, the α-(1→2)-mannosyltransferases CmsA and CmsB (also known as Ktr4 and Ktr7, respectively) were shown to biosynthesize the α-core-mannan of FTGM (15–17). In Δ*cmsA* and/or Δ*cmsB* strains, hyphal elongation was remarkably suppressed and conidium formation was defective (15, 16). Moreover, the Δ*ktr4* mutant was significantly less virulent than the parental strain (16). These data indicate that the formation of the α-core-mannan of FTGM is important for mycelial growth, conidium formation, and virulence in mice. Among the α-core-mannan biosynthetic enzymes, mannosyltransferases involved in the biosynthesis of α-(1→6)-mannosyl residues are expected to be as important as CmsA and CmsB; however, such mannosyltransferases remain unidentified.

In Saccharomyces cerevisiae, except for the endoplasmic reticulum-localized glycosyltransferases (GTs) involved in N-glycan and GPI anchor biosynthesis, 3 GT families containing α-(1→6)-mannosyltransferases are known: GT32, comprising Och1p and Hoc1p; GT34, comprising Mnn10p and Mnn11p; and GT62, comprising Mnn9p, Van1p, and Anp1p (18). Och1p is the initial enzyme for N-glycan outer chain biosynthesis in S. cerevisiae (19). Van1p and Mnn9p form a complex, M-Pol I, while Mnn9p, Anp1p, Mnn10p, Mnn11p, and Hoc1p form M-Pol II, both of which elongate the N-glycan outer chain in the yeast (20–23). In the pathogenic yeast Cryptococcus neoformans, Hoc1p and Hoc3p have been reported to be involved in synthesizing the α-(1→6)-mannosyl residues of O-glycans (24). Recently, an Och1-like enzyme (OchC) was reported to be responsible for biosynthesizing the α-(1→6)-mannosyl residues of Af3c, a zwitterionic glycolipid in A. fumigatus (25). Overall, homologous glycosyltransferases seem capable of synthesizing different glycans in different species, despite showing the same enzymatic activity *in vitro*. In a previous study, nine α-(1→6)-mannosyltransferases from A. fumigatus were isolated as S. cerevisiae homologs (26). Henry et al. (26) constructed 11 gene disruption strains, targeting these 9 genes plus 2 putative α-(1→2)-mannosyltransferase genes, and showed that the disrupted strains produced less mannan in the conidia. They characterized multiple gene disruption strains, but did not describe the phenotypes of individual strains (26). In addition, they performed monosaccharide analysis on the fractionated cell wall components based on differences in solubility in alkaline solutions, but a more precise analysis, such as nuclear magnetic resonance (NMR) analysis, was not conducted (26). Therefore, we hypothesized that the α-(1→6)-mannosyltransferases involved in the biosynthesis of the α-(1→6)-mannosyl residues of α-core-mannan are among these nine α-(1→6)-mannosyltransferase candidates.

The aim of this study was to identify the α-(1→6)-mannosyltransferases involved in synthesizing the α-core-mannan of FTGMs. We analyzed the 9 gene disruptants in A. fumigatus. NMR analysis revealed that the Δ*anpA* strain did not produce the α-core-mannan chain of FTGM. We also showed that Escherichia coli recombinants of AnpA exhibited α-(1→6)-mannosyltransferase activity *in vitro*. Molecular modeling, docking, and molecular dynamics (MD) simulations revealed that the structure of AnpA contributes to α-(1→6)-mannoside bond formation at the α-core-mannan of FTGMs, and the C6-hydroxyl group of α-methyl mannoside can act as a model acceptor substrate for GDP mannose (GDP-Man) at the active site of AnpA.

## RESULTS

### Candidate enzyme selection of α-(1→6)-mannosyltransferases responsible for the biosynthesis of the α-core-mannan of FTGM in A. fumigatus.

Based on our hypothesis, we selected the nine α-(1→6)-mannosyltransferases of A. fumigatus listed in the study by Henry et al. (26): Van1 (AFUB_031580), Mnn9 (AFUB_018530), Anp1 (AFUB_063940), Mnn10 (AFUB_067830), Mnn11 (AFUB_030560), Och1-1 (AFUB_056120), Och1-2 (AFUB_000710), Och1-3 (AFUB_084570), and Och1-4 (AFUB_079410). The sequence at the C-terminus of Mnn9 (AFUB_018530) is incorrect and has been corrected by Henry et al. (26). The revised sequence was used in this study (26).

**FIG 1:**
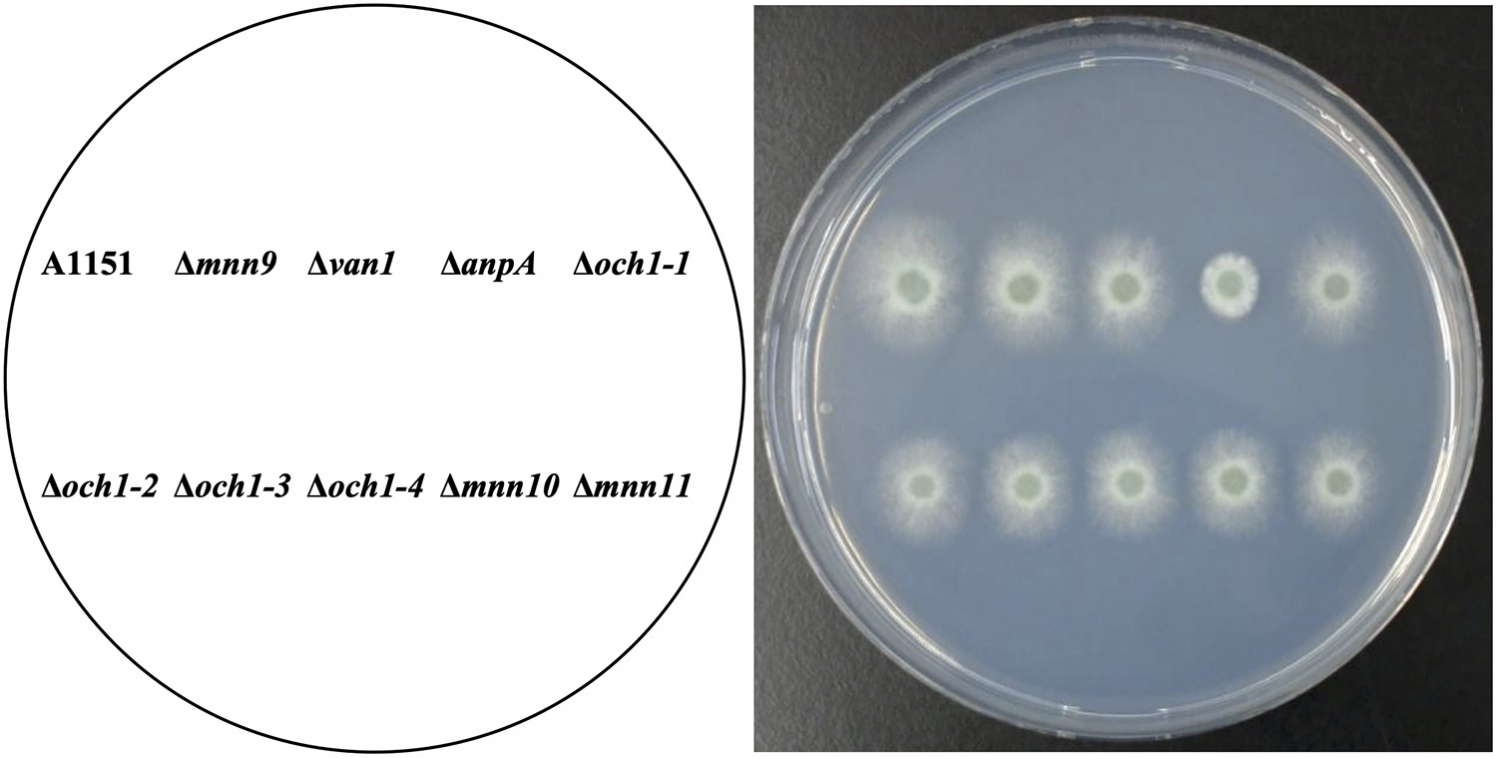
Analysis of putative α-(1→6)-mannosyltransferase genes in Aspergillus fumigatus. Colony morphology of putative α-(1→6)-mannosyltransferase gene disruptants cultured on minimal medium (MM) agar at 37°C for 2 days. The agar medium was inoculated with 1.0 × 10^4^ conidiospores.

First, we constructed single gene disruptants and observed their colony phenotype. Figure 1 shows the morphology of colonies grown on MM at 37°C for 2 days (Fig. 1 and Fig. S1). While most gene disruptants did not show significant changes compared with the wild-type, the Δ*AFUB_063940* strain displayed decreased mycelial elongation and reduced conidia formation—the similar phenotypes observed after disrupting *cmsA* or *cmsB*, both of which participate in the biosynthesis of the α-core-mannan. This result suggests that AFUB_063940 may also be involved in FTGM biosynthesis. AFUB_063940 will further be referred to as *anpA*, according to the Aspergillus gene nomenclature recommendations, but has also been previously called *ANP1*, following Saccharomyces gene nomenclature (26).

10.1128/msphere.00484-22.1 FIG S1 Construction of A. fumigatus strains used in this study. (A) Chromosomal maps of the gene-disrupted strains of *mnn9*, *van1*, *anpA*, *och1*, *och2*, *och3*, *och4*, *mnn10*, and *mnn11*. The positions of the primers are indicated by arrows. *pyrG*, orotidine 5′-phosphate decarboxylase gene from Aspergillus nidulans. (B) Confirmation of correct recombination of each strain using PCR analysis. Electrophoretic analyses of products amplified by PCR using primer pairs xxxX-F/xxxX-R. M, DNA size markers (Gene Ladder Wide 2; Nippon Gene, Tokyo, Japan). Download FIG S1, PDF file, 0.6 MB. Copyright © 2022 Kadooka et al. 2022 Kadooka et al. https://creativecommons.org/licenses/by/4.0/ This content is distributed under the terms of the Creative Commons Attribution 4.0 International license.

### Δ*anpA* strain phenotypes.

AnpA is a member of the GT62 family that has an amino acid sequence homology of 40.0% (in the 120 to 300 amino acid region) and 51.5% (in the 150 to 291 amino acid region) with Mnn9 and Van1 (other members of the GT62 family), respectively (Fig. S2). Because AnpA, Mnn9, and Van1 belong to the same GT62 family, a detailed phenotypic analysis was performed to determine the differences between the Δ*anpA*, Δ*mnn9*, and Δ*van1* strains. We observed their colony formation at 30°C, 37°C, 42°C, and 50°C for 2 days. Δ*mnn9* and Δ*van1* strains formed colonies similar to those of the A1151 strain under these conditions but the Δ*anpA* strain showed growth defects at 37°C and 42°C (Fig. S3), suggesting that AnpA functions differently from the other two GT62 members *in vivo*.

10.1128/msphere.00484-22.2 FIG S2 Multiple sequence alignment of Mnn9p, AnpA, Mnn9, and Van1 amino acid sequences. Well-preserved residues are highlighted by blue boxes. Fully conserved amino acid residues are indicated by white letters on a red background. Aligned amino acid residues with similar properties are indicated in red. Arrows indicate amino acid residues required for the coordination of manganese ions. Download FIG S2, PDF file, 0.7 MB. Copyright © 2022 Kadooka et al. 2022 Kadooka et al. https://creativecommons.org/licenses/by/4.0/ This content is distributed under the terms of the Creative Commons Attribution 4.0 International license.

10.1128/msphere.00484-22.3 FIG S3 Phenotypic analysis of GT62 family mutants. The colony morphology of A1151, Δ*mnn9*, Δ*van1*, and Δ*anpA* strains, cultured on MM agar at 30°C, 37°C, 42°C, 50°C for 2 days, respectively. The agar medium was inoculated with 1.0 × 10^4^ conidiospores. Download FIG S3, PDF file, 0.6 MB. Copyright © 2022 Kadooka et al. 2022 Kadooka et al. https://creativecommons.org/licenses/by/4.0/ This content is distributed under the terms of the Creative Commons Attribution 4.0 International license.

**FIG 2:**
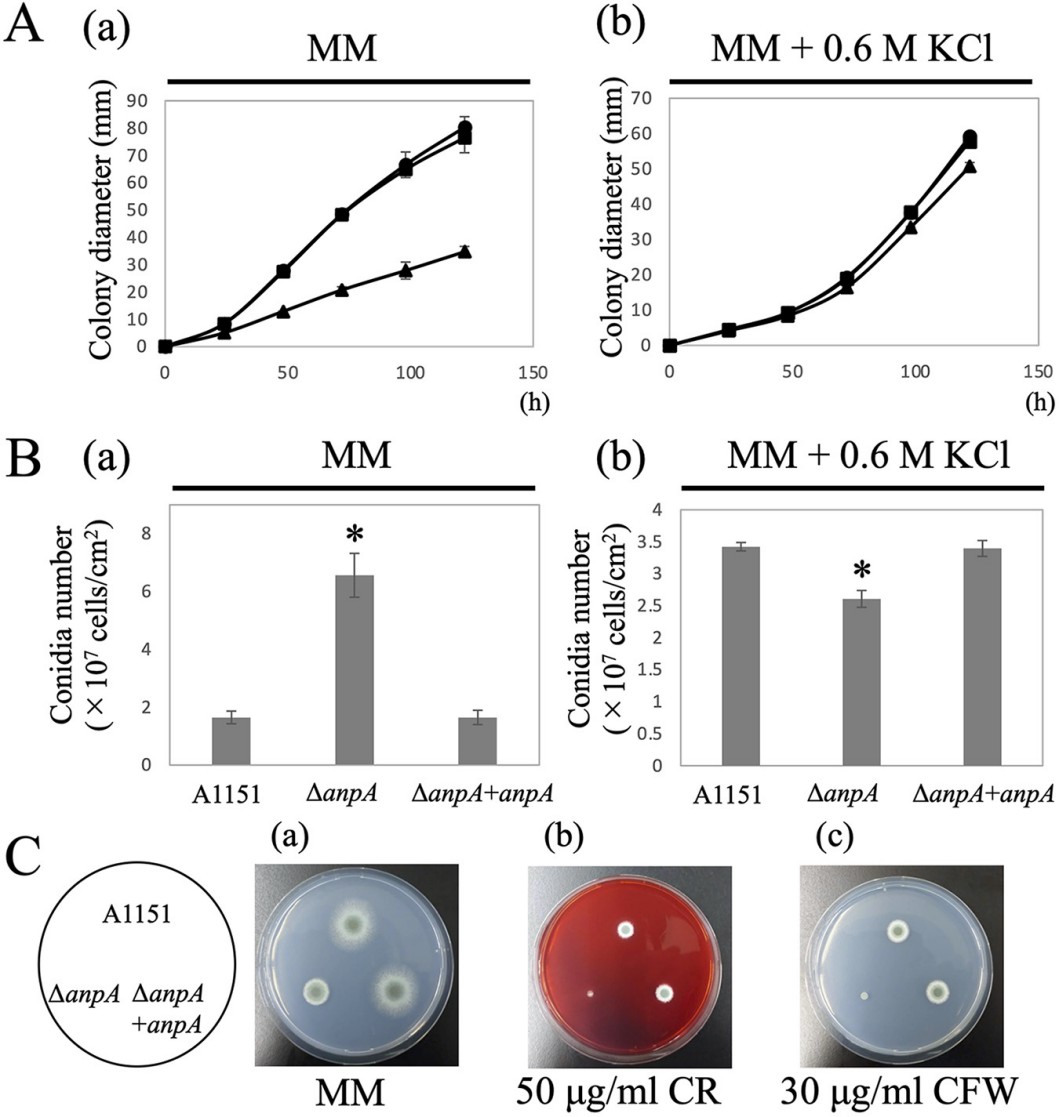
Phenotypic analysis of GT62 family gene mutants. (A) Colony diameters of the A1151 (circle), Δ*anpA* (triangle), and Δ*anpA*+*anpA* (square) strains when cultured on MM agar (a) or MM agar supplemented with 0.6 M KCl (b) at 37°C for 0, 24, 48, 72, 96, and 120 h. (B) Number of conidia per colony area in the A1151, Δ*anpA*, and Δ*anpA*+*anpA* strains cultured on MM agar (a) and MM agar supplemented with 0.6 M KCl (b) at 37°C for 5 days. Asterisks indicate a statistically significant difference compared to the A1151 strain. *, *P* < 0.001 by ANOVA. (C) Sensitivity to cell wall stress inducers Congo red (CR) and calcofluor white (CFW). The A1151, Δ*anpA*, and Δ*anpA*+*anpA* strains were grown on MM agar (a) supplemented with 50 μg/mL of CR (b) or 30 μg/mL of CFW (c) at 37°C for 2 days.

To confirm that the Δ*anpA* phenotype was truly due to the disruption of the *anpA* gene (Fig. S4), we constructed an *anpA* gene complementation strain (Δ*anpA*+*anpA*) and measured the hyphal elongation rate and the conidia number per colony area (Fig. 2A and B). The Δ*anpA*+*anpA* strain showed colony morphology similar to the A1151 strain on MM (Fig. 2C, panel [a]). The diameters of the Δ*anpA* colonies were 0.6-, 0.46-, 0.4-, 0.38-, and 0.43-fold smaller than those of the A1151 colonies when cultured on MM at 37°C for 24, 48, 72, 96, and 122 h, respectively (Fig. 2A, panel a). The result suggests that A. fumigatus AnpA plays a significant role in hyphal elongation. By contrast, the conidia number per colony area of the Δ*anpA* strain was 4.0-fold higher compared with that of the A1151 strain after 122 h (Fig. 2B, panel a). The growth defects of the Δ*anpA* strain were remedied under high osmotic support conditions (MM supplemented with 0.6 M KCl) (Fig. 2A, panel b), but their conidia number per colony area reduced by 0.76-fold compared with that of the A1151 strain (Fig. 2B, panel b).

10.1128/msphere.00484-22.4 FIG S4 Complementation of *anpA* for Δ*anpA*. (A) Schematic representation of Δ*anpA* complementation with *anpA*. Primer positions are indicated by arrows. *pyrG*, orotidine 5′-phosphate decarboxylase gene from Aspergillus nidulans; *hph*, hygromycin B phosphotransferase gene. (B) Confirmation of the correct recombination of *anpA* using PCR analysis. Results of electrophoretic analysis of products amplified by PCR using the primer pairs anpA-F/anpA-R are shown. M, DNA size markers (Gene Ladder Wide 2). Download FIG S4, PDF file, 0.1 MB. Copyright © 2022 Kadooka et al. 2022 Kadooka et al. https://creativecommons.org/licenses/by/4.0/ This content is distributed under the terms of the Creative Commons Attribution 4.0 International license.

Next, we investigated the effect of *anpA* disruption on cell wall integrity by testing the drug sensitivities of A1151, Δ*anpA*, and Δ*anpA*+*anpA* strains to the cell wall stress inducers Congo red (CR, β-glucan synthesis inhibitor) (Fig. 2C, panel b) and calcofluor white (CFW, chitin synthesis inhibitor (Fig. 2C, panel c). All strains were cultured on MM supplemented with 50 μg/mL CR or 30 μg/mL CFW at 37°C for 2 days. The Δ*anpA* strain showed a higher sensitivity to CR and CFW compared with the other 2 strains (Fig. 2C, panels b and c), implying that *anpA* disruption causes abnormalities in the cell wall structure like modified β-glucan and/or chitin contents due to altered cell wall mannan structures in A. fumigatus.

### Phylogenetic analysis of α-(1→6)-mannosyltransferases belonging to the GT62 family in yeast and fungi.

**FIG 3:**
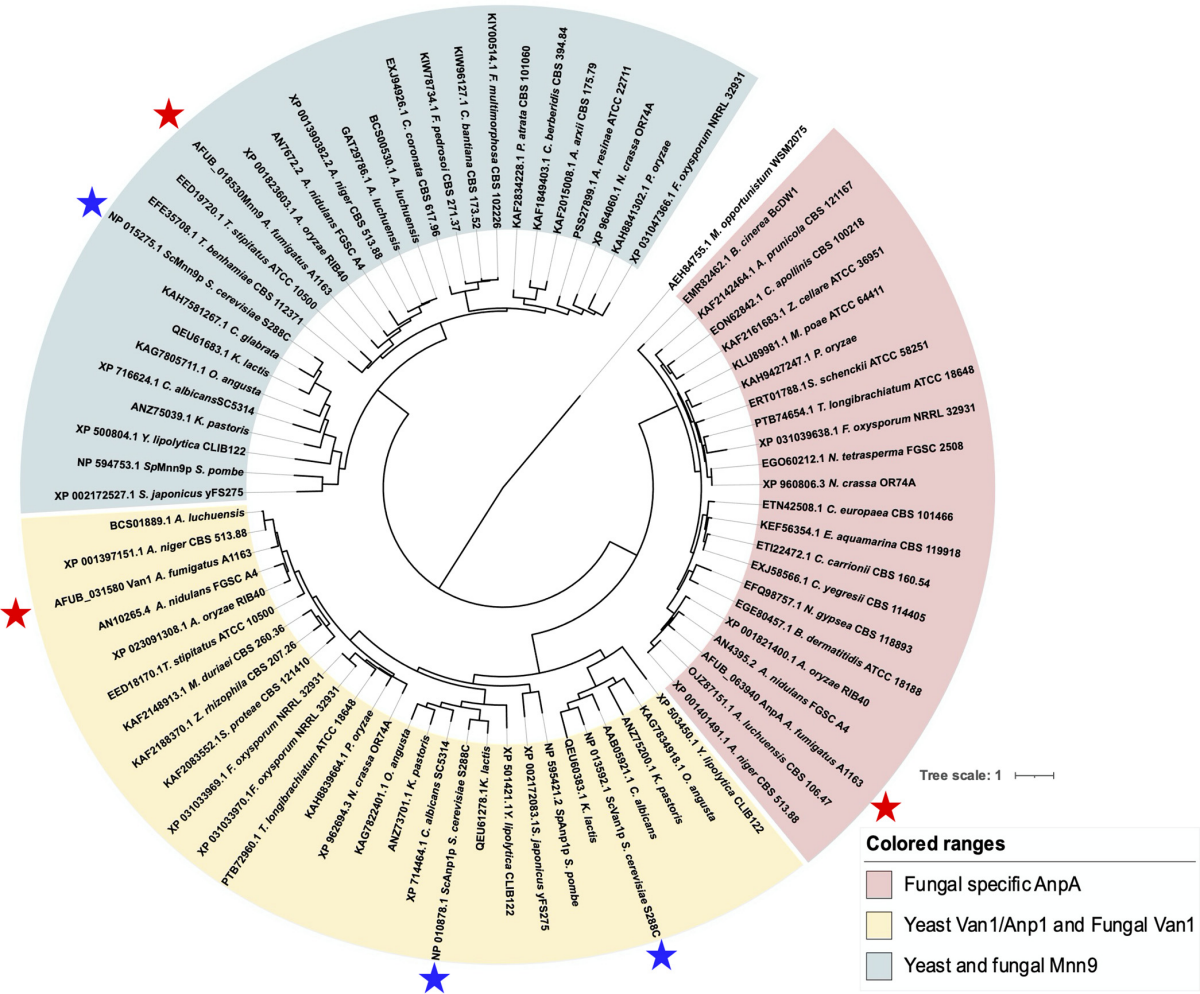
Phylogenetic analysis of glycosyltransferase 62 (GT62) family proteins from yeast and filamentous fungi strains. Protein sequences were downloaded from the NCBI or FungiDB. The phylogenetic tree was drawn with iTOL, and the alignment and phylogenetic tree inference were performed with MAFFT and RAxML included in ETE v3. The glycosyltransferase 62 family proteins from Saccharomyces cerevisiae S288C and A. fumigatus A1163 are marked with blue and red stars. AnpA of A. fumigatus A1163 is clearly classified into an independent fungal-specific AnpA clade, separate from the yeast Van1/Anp1 and the fungal Van1 clade.

The sequences of GT62 proteins from a wide range of yeasts and fungi were used to construct an evolutionary phylogenetic tree (Fig. 3). The data set for analysis was obtained by NCBI Protein BLAST (https://www.ncbi.nlm.nih.gov/) or FungiDB (https://fungidb.org) using the amino acid sequences of S. cerevisiae Van1p, Mnn9p, and Anp1p as search queries. The Mnn9p homologous protein from a rhizobia Mesorhizobium opportunistum WSM2075(T) (27), presumably with a common ancestor, was used as the outgroup. GT62 mannosyltransferases were segregated into 3 clades: a fungal-specific AnpA clade, a yeast and fungal Mnn9 clade, and a clade containing yeast Van1p/Anp1p and fungal Van1 (Fig. 3). Some GT62 proteins from filamentous fungi, including AnpA, formed an independent fungus-specific clade that did not contain any yeast-derived proteins (Fig. 3). S. cerevisiae Anp1p was phylogenetically closer to S. cerevisiae Van1p and A. fumigatus Van1 than A. fumigatus AnpA, which was classified under the fungal-specific AnpA group (Fig. 3). The data clearly show that S. cerevisiae Anp1p and A. fumigatus AnpA are phylogenetically distinct despite their similar names, strongly suggesting that they have distinct functions. Furthermore, these results indicate that AnpA is involved in the biosynthesis of fungus-specific glycans, such as FTGM.

### FTGM structures of A1151, Δ*mnn9*, Δ*van1*, Δ*anpA*, and Δ*anpA*+*anpA* strains.

**FIG 4:**
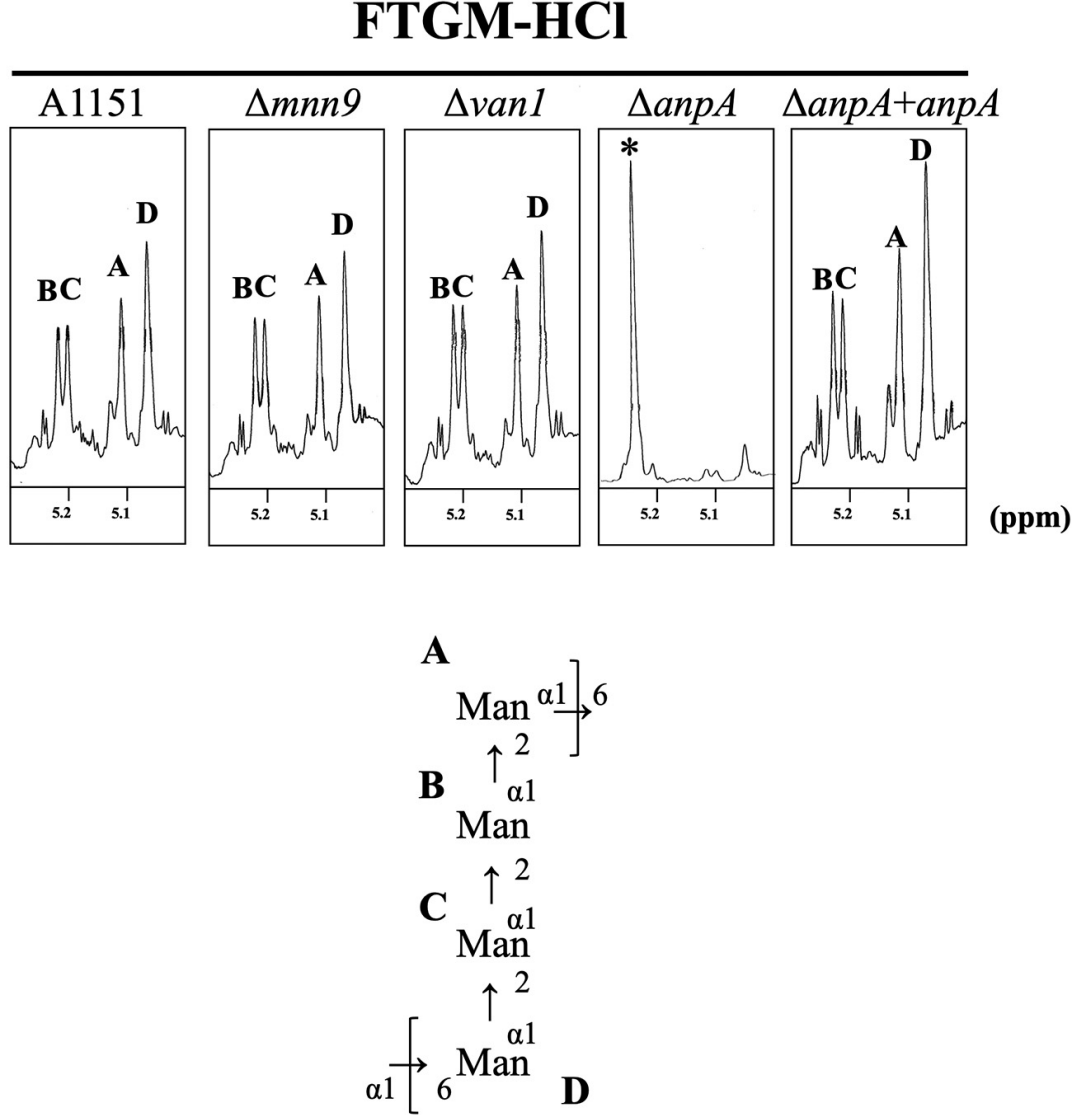
AnpA is essential for the core-mannan structure of fungal-type galactomannan (FTGM). Proton nuclear magnetic resonance (^1^H-NMR) analysis of the core-mannan of FTGM, with the galactofuran side chains removed (FTGM-HCl), from wild-type (WT, A1151), Δ*mnn9*, Δ*van1*, Δ*anpA*, and Δ*anpA*+*anpA* strains. Regions corresponding to the mannose-derived H-1 signal are shown. The chemical shifts in the H-1 signal corresponding to the core-mannan structure are summarized in Table 1. Asterisks indicate unidentified NMR signals (15). The proton chemical shifts were referenced relative to internal acetone at δ 2.225 ppm.

To clarify the role of AnpA in the biosynthesis of the FTGM α-core-mannan, we investigated the FTGM structures. The involvement of Mnn9 and Van1, members of the GT62 family, in core-mannan biosynthesis was also investigated. The total GM fraction was extracted and purified from all the strains and treated with 0.1 M HCl to remove the β-(1→5)-/β-(1→6)-galactofuran side chains from FTGM and OMGM. OMGMs were removed from the total GM fraction using the β-elimination method to obtain the purified FTGM fraction, whose α-core-mannan structures were analyzed using ^1^H-NMR spectroscopy. The 4 signals of A1151, Δ*mnn9*, Δ*van1*, and Δ*anpA*+*anpA* strains indicate the H-1 signal of the chemical shift of the α-(1→2)-/α-(1→6)-mannan (Fig. 4 and Table 1) (15). However, these α-core-mannan signals were absent in the ^1^H-NMR spectrum of the Δ*anpA* strain (Fig. 4), which was similar to the spectral patterns of Δ*cmsA* and Δ*cmsB*, (15) indicating that the FTGM α-core-mannan was also lost in Δ*anpA*. On the other hand, no spectral changes were observed due to the disruption of *van1* or *mnn9* (Fig. 4 and Table 1), establishing that these 2 GTs were not involved in the synthesis of the FTGM α-core-mannan.

**TABLE 1:** ^1^H chemical shift value obtained for FTGM core-mannan^*a*^

Sugar residue	A1151	Δ*van1*	Δ*mnn9*	Δ*anpA*	Δ*anpA::anpA*
→6Manα1→2Manα1→2Manα1→2Manα1→6	5.057	5.057	5.059	-	5.052
→6Manα1→2Manα1→2Manα1→2Manα1→6	5.216	5.216	5.216	-	5.212
→6Manα1→2Manα1→2Manα1→2Manα1→6	5.234	5.234	5.236	-	5.231
→6Manα1→2Manα1→2Manα1→2Manα1→6	5.108	5.108	5.108	-	5.103

*^a^*H-1 signal, δ (ppm).

Next, we evaluated the involvement of AnpA in the biosynthesis of terminal-mannosyl residues of N-glycan in A. fumigatus (Fig. S5). A 3xFLAG-tagged SucA (Afu2g01240/AFUB_018320), an invertase highly modified with N-glycans in A. fumigatus (28), was expressed in the A1151 and Δ*anpA* strains and detected by immunoblotting to estimate the length of N-glycan (Fig. S5). As SucA has 9 potential N-glycosylation sites, a lower apparent molecular weight should be observed in the Δ*anpA* strain than in the A1151 strain if AnpA contributes to N-glycan elongation. The apparent molecular weight of SucA expressed in Δ*anpA* was comparable to SucA expressed in A1151 (Fig. S5). This finding clearly demonstrates that AnpA does not contribute to the elongation of N-glycan mannosyl residues.

10.1128/msphere.00484-22.5 FIG S5 Detection of invertase expression in A1151 and Δ*anpA* cells. Invertase (SucA) was overexpressed using the pPTR-II-SucA plasmid in the A1151 and Δ*anpA* strains. Extracted proteins were immunoblotted using anti-FLAG polyclonal antibodies. Download FIG S5, PDF file, 0.07 MB. Copyright © 2022 Kadooka et al. 2022 Kadooka et al. https://creativecommons.org/licenses/by/4.0/ This content is distributed under the terms of the Creative Commons Attribution 4.0 International license.

### AnpA has *in vitro* α-(1→6)-mannosyltransferase activity.

**FIG 5:**
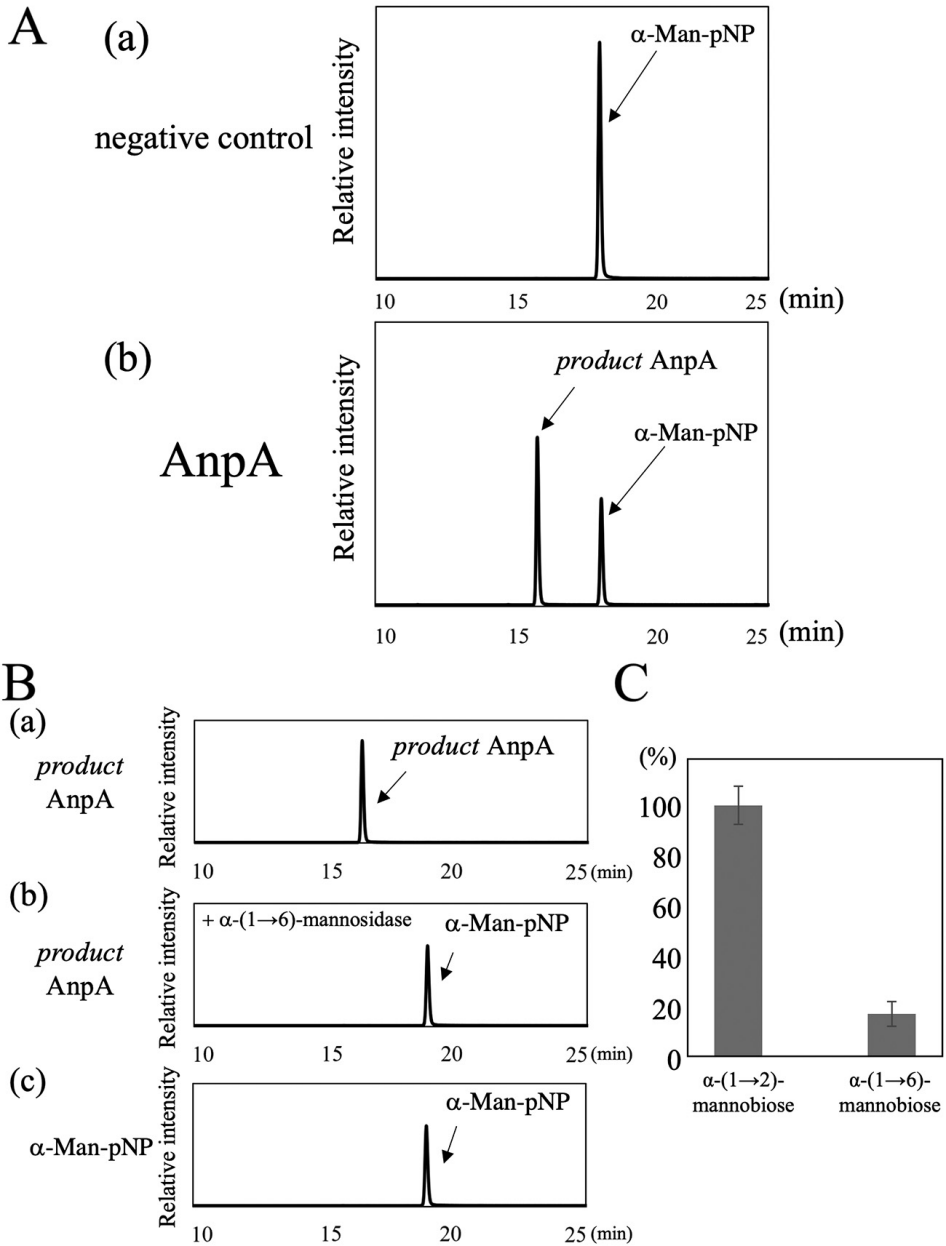
Mannosyltransferase activity of AnpA. (A) Chromatograms of AnpA mannosyltransferase activity assays using *p*-nitrophenyl α-d-mannopyranoside (α-Man-pNP) as the artificial acceptor substrate. A reaction mixture (40 μL) containing 50 mM HEPES-NaOH (pH 6.8), 100 mM NaCl, 30 mM KCl, 5% glycerol, 1 mM MnCl_2_, 1.5 mM α-Man-pNP (acceptor substrate), 5 mM GDP (GDP)-Man (donor substrate), and 7.2 μg of purified AnpA was incubated at 30°C for 16 h. Chromatograms show typical results of the assay without AnpA (negative control, [a]) and with AnpA (b). The reaction without AnpA yielded only peaks derived from the α-Man-pNP at 18.2 min, whereas in fractions with AnpA, a reaction product (termed *product*) was observed at 16.0 min. (B) Structural analysis of *product* using substrate-specific mannosidases. A chromatogram shows the purified *product* AnpA (a) and α-Man-pNP (c). The purified *product* AnpA was reacted with α-(1→6)-mannosidase (b). *Product* AnpA could react with α-(1→6)-mannosidase and was digested to α-Man-pNP. (C) Substrate specificity of AnpA. A reaction mixture (40 μL) containing 50 mM HEPES-NaOH (pH 6.8), 100 mM NaCl, 30 mM KCl, 5% glycerol, 1 mM MnCl_2_, 5 mM GDP-Man (donor substrate), 7.44 μg of purified AnpA, and 2.5 mM α-(1→2)-mannobiose or α-(1→6)-mannobiose (acceptor substrates) was incubated at 30°C for 6 h. Each reaction mixture was labeled with ethyl 4-aminobenzoate (ABEE) and then analyzed using high-performance liquid chromatography (HPLC). The 100% value corresponds to the synthesis of 8.4 nmol of α-Man-(1→6)-α-Man-(1→2)-α-Man-ABEE using 1 mg of AnpA per minute.

To characterize the enzymatic activity of AnpA, we prepared recombinant AnpA using the bacterial expression system. We measured mannosyltransferase activity at 30°C for 16 h using the purified recombinant AnpA (0.1 μg/μL), α-Man-pNP (1.5 mM) as a sugar acceptor, GDP-Man (5 mM) as a sugar donor, and Mn^2+^ (1.0 mM) as a cofactor. The reaction mixtures were separated and analyzed by reverse-phase HPLC using a C18 column and an UV detector. The fraction without AnpA exhibited only α-Man-pNP peak at 18.2 min (Fig. 5A, panel a); however, fractions with AnpA showed a new peak (defined *product* AnpA) at 16 min (Fig. 5A, panel b).

To determine the chemical structure of the product, it was collected (Fig. 5B, panel a) and digested by α-(1→6)-specific mannosidase from *Xanthomonas manihotis*. The product was completely digested and converted to α-Man-pNP (Fig. 5B, panel b and c), certifying that the product of AnpA was α-Man-(1→6)-α-Man-pNP and verifying that AnpA possessed α-(1→6)-mannosyltransferase activity *in vitro*. These results strongly support the hypothesis that AnpA is responsible for transferring the α-(1→6)-mannosyl residue in the FTGM α-core-mannan.

### Substrate specificity of AnpA.

To explore the functional differences between AnpA and the other α-(1→6)-mannosyltransferases, we measured its activity on 2 acceptor substrates: α-(1→6)-mannobiose and α-(1→2)-mannobiose. AnpA’s mannosyltransferase activity toward α-(1→6)-mannobiose was 16.9% relative to that toward α-(1→2)-mannobiose (100%) (Fig. 5B, panel c). This substrate specificity of AnpA is unlike that of the yeast α-(1→6)-mannan polymerase complex comprising Mnn9p and Van1p, the other GT62 family members (20–23) and is consistent with the activity required for α-core-mannan biosynthesis: the transfer of mannose to the hydroxyl group at position 6 at the nonreducing end of α-(1→2)-mannotetraose.

### AnpA 3D structure prediction.

Among GT62 α-(1→6)-mannosyltransferase homologs, only 1 crystal structure – Mnn9p from S. cerevisiae – has been reported (29). The structure shows that Mnn9p is a homodimer, with Mn^2+^ and GDP bound to the active site, but detailed information on its interaction and reaction mechanism with GDP-Man and the acceptor substrate is lacking. AlphaFold2 successfully predicted the 3D structure of the catalytic domain of AnpA with a high pLDDT score of 91.2 and no Ramachandran outliers (Fig. S6A) (30). AnpA was predicted to form a homodimer and to have a mixed α/β fold with 13 β-strands and 11 α-helices containing the core nine-stranded β-sheet surrounding the α-helices (Fig. S6B), just like the reported structure of S. cerevisiae Mnn9p (Fig. S6C) (29). In the crystal structure of Mnn9p, its Mn^2+^ ion is coordinated by Asp238 and His389, with the phosphate group of GDP (Fig. S6D). The first Asp in the metal-binding DXD motif, which is typical in GT-A, has been artificially mutated to Asn236 and does not participate in binding to Mn^2+^ (Fig. S6D). In the predicted structure of AnpA, Asp244 and Asp246 of the DXD motif are conserved and presumed to bind Mn^2+^ together with His395 (Fig. S6E). In addition, the binding pocket for the guanosine moiety was also inferred, but some amino acid residues were substituted from Mnn9p (Fig. S6D and E).

10.1128/msphere.00484-22.6 FIG S6 The dimeric structure of the catalytic domain of AnpA as predicted by AlphaFold2. (A) A ribbon representation of the AnpA dimer is colored from blue (high confidence) to red (low confidence) as per the pLDDT score. The N-termini and C-termini are labeled. Except for the C-termini, the model had high pLDDT score (mean = 91.2). (B) The predicted overall structure of the AnpA dimer. Secondary structures are color-coded red:helix, yellow:strand, and green:loop. AnpA was predicted to have a mixed α/β fold with 13 β-strands and 11 helices in a monomer. (C) The crystal structure of Mnn9p from S. cerevisiae. (D) The metal and GDP-Man binding site of Mnn9p from S. cerevisiae. The Mn^2+^ ion (magenta), GDP, and the binding residues are shown by ball-and-stick models and labeled. (E) The putative metal and GDP-Man binding site of AnpA. The residues are shown by stick models and labeled. Download FIG S6, PDF file, 1.1 MB. Copyright © 2022 Kadooka et al. 2022 Kadooka et al. https://creativecommons.org/licenses/by/4.0/ This content is distributed under the terms of the Creative Commons Attribution 4.0 International license.

### Docking and MD simulations of the AnpA–substrate complex.

**FIG 6:**
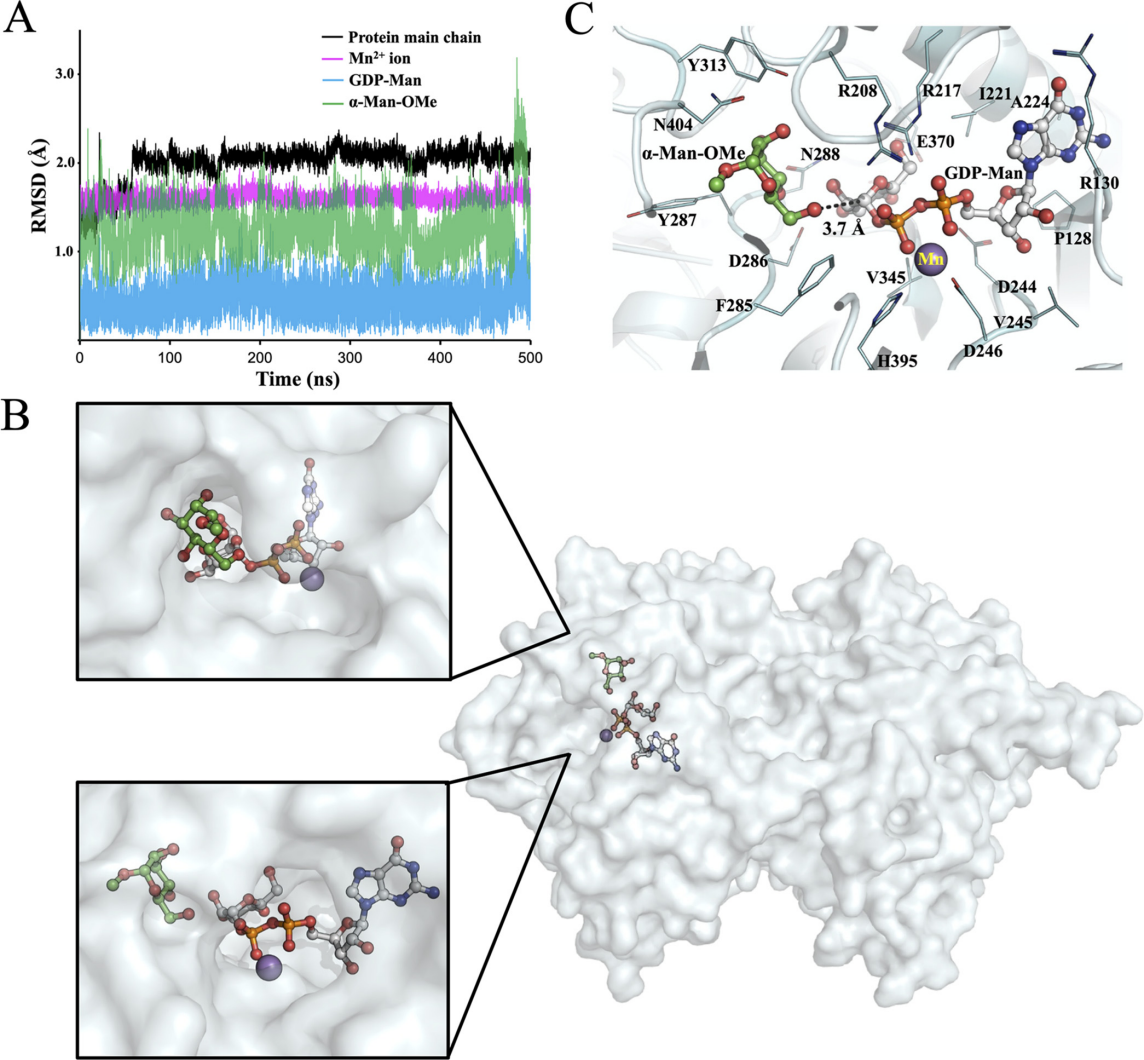
Representative structure of AnpA in complex with GDP-Man and α-Man-OMe from the most populated cluster of the molecular dynamics (MD) simulation and root mean square deviation (RMSD) analysis. (A) RMSD analysis of the main amino acid chain (black), Mn^2+^ ion (magenta), GDP-Man (cyan) as the donor substrate, and α-Man-OMe (green) as the acceptor substrate during a 500-ns MD simulation. (B) Molecular surface representation of AnpA in complex. The Mn^2+^ ion (magenta), GDP-Man (gray), and α-Man-OMe (green) are shown as ball-and-stick models. The two windows are enlarged views of the active site from two directions. GDP-Man and α-Man-OMe were bound in positions where they could directly interact. (C) Stick model representation of the substrates bound to the active site. The residues are shown by thin stick models and labeled. The Mn^2+^ ion was retained in the active site by interacting with Asp246, His395, and two phosphate oxygens in the GDP moiety. The binding of α-Man-OMe (green) was stabilized by hydrogen bonds with Asp286 and Asn190, and van der Waals interactions with Phe285, Tyr287, Tyr313, and Asn404. The dashed line represents the mean distance (3.7 Å) between the 6-OH of α-Man-OMe and the 1-C of the mannose moieties of GDP-Man (gray).

To investigate the binding of Mn^2+^ and the substrates to AnpA and the mechanism with which it forms an α-(1→6)-glycosidic bond, we performed MD simulations analyses using its substrates and the predicted structure of AnpA. The structure of AnpA, in complex with the Mn^2+^ ion, the donor substrate GDP-Man, and the acceptor substrate α-Man-OMe, was generated by docking simulations using the GNINA program (31). In addition, an unrestrained 500 ns MD simulation was performed to accommodate the active site substrates and test the stability of the complex (Fig. 6A). The root mean square deviations (RMSDs) of the protein main chain, Mn^2+^, and the substrates between each frame of the MD simulation and the initial structure are shown in Fig. 6A. Although the RMSD of the protein main chain reached 2.0 Å during the first 50 ns, all RMSDs remained below 3.0 Å, indicating that the overall complex was sufficiently stable (Fig. 6A). Anticipating sufficient equilibration, further analyses were performed using the data from 65 to 500 ns of the MD simulation. The RMSD-based cluster analysis of the protein main chain atoms also clearly illustrated the interactions in the complex. Fig. 6B and 6C show the representative AnpA structure of the most populated cluster (53% population) obtained with an RMSD cutoff 1.0 Å. During the MD simulation, Mn^2+^, GDP-Man, and α-Man-OMe were retained inside the AnpA molecule. The Mn^2+^ atom was bonded by Asp246, His395, and two phosphate oxygens from GDP. The phosphate group of GDP also interacted with Arg208 and Arg217. The guanosine moiety formed van der Waals interactions with Pro128, Arg130, Ala224, and Ile221, and a hydrogen bond with the main chain carbonyl of Leu158 (Fig. 6C and Fig. S6E). Although these interacting amino acid residues were not completely conserved between AnpA and S. cerevisiae Mnn9p (Fig. S6D and E), the MD simulation showed that AnpA stably bound Mn^2+^ and GDP-Man at its active site with a similar orientation to that found in the Mn^2+^/GDP complex of S. cerevisiae Mnn9p.

The binding of α-Man-OMe to the active site appeared to be stabilized by hydrogen bonds formed with Asp286 and Asn288, the main chain atoms of Tyr287 and Glu311, and van der Waals interactions with Phe285, Tyr287, Tyr313, and Asn404 (Fig. 6C). While the RMSD of the acceptor substrate fluctuated the most, it continuously interacted with the donor substrate at the active site of AnpA. We analyzed the fluctuations in the distance between α-Man-OMe and GDP-Man using MD simulation data from 65 to 500 ns. The C6-hydroxyl group (6-OH) of α-Man-OMe was located closest to GDP-Man, and the average distance between the 6-OH oxygen and the mannose site 1-C of GDP-Man was 3.72 ± 0.66 Å in the MD simulation, indicating that the donor and acceptor groups directly interact in an orientation suitable for the formation of an α-(1→6)-glycosidic bond (Fig. 6B and 6C).

In the complex obtained by MD simulation, α-Man-OMe was about 10 Å inside from the molecular surface of AnpA (Fig. S7A), indicating that the terminal mannose residue of a physiological acceptor substrate recognized by AnpA must penetrate to this position. This also implies that the acceptor substrate recognized by AnpA *in vivo* must be the end of a linear mannan chain of some length (Fig. S7B).

10.1128/msphere.00484-22.7 FIG S7 AnpA requires longer sugar chains as its physiological acceptor substrate. (A) In the complex obtained by MD simulation, the position of α-Man-OMe, which was used as a acceptor substrate model, was about 10 Å inside from the molecular surface of AnpA. The Mn^2+^ ion (magenta), GDP-Man (gray), and α-Man-OMe (green) are shown by ball-and-stick models. (B) The energy-minimized conformation of α-Man-(1→2)-α-Man-(1→2)-α-Man-(1→2)-α-Man-OMe was superimposed on the simulated complex structure. When the nonreducing terminal Man was superimposed on the α-Man-OMe of the complex, the reducing terminal residue reached the molecular surface. Therefore, glycans as long as α-Man-(1→2)-α-Man-(1→2)-α-Man-(1→2)-α-Man would be required as the physiological acceptor substrates of AnpA *in vivo*. Download FIG S7, PDF file, 0.4 MB. Copyright © 2022 Kadooka et al. 2022 Kadooka et al. https://creativecommons.org/licenses/by/4.0/ This content is distributed under the terms of the Creative Commons Attribution 4.0 International license.

### Pathogenicity of A1151, Δ*anpA*, and Δ*anpA*+*anpA* strains.

Since disruption of *CmsA*/*ktr4* has been reported to reduce the virulence of A. fumigatus (16), FTGM may play an important role in the pathogenicity of the fungus. Therefore, we investigated the effect of *anpA* disruption on virulence. The C57BL/6 bloodline mice were injected with cyclophosphamide/cortisone acetate to suppress immunity. The cohort infected with the Δ*anpA* strain showed survival similar to those infected with the A1151 and Δ*anpA*+*anpA* strains, with no significant differences in virulence. (Fig. S8). This result suggests that AnpA is essential for FTGM biosynthesis, but unlike CmsA, not important for pathogenicity.

10.1128/msphere.00484-22.8 FIG S8 Virulence assay of A1151, Δ*anpA*, and Δ*anpA*+*anpA* strains in an invasive aspergillosis mouse model. The C57BL/6 bloodline mice were injected with cyclophosphamide/cortisone acetate to suppress immunity, and intranasally inoculated with 40 μL of phosphate-buffered saline containing 1.5 × 10^5^ conidia per mouse. Download FIG S8, PDF file, 0.06 MB. Copyright © 2022 Kadooka et al. 2022 Kadooka et al. https://creativecommons.org/licenses/by/4.0/ This content is distributed under the terms of the Creative Commons Attribution 4.0 International license.

## DISCUSSION

In this study, we identified the α-(1→6)-mannosyltransferase responsible for biosynthesis of the FTGM α-core-mannan structure in A. fumigatus. Phylogenetic analysis revealed that AnpA belongs to the fungal-specific AnpA clade, a unique clade formed by proteins homologous to AnpA in Pezizomycotina (Fig. 3). Fungal AnpA orthologs are phylogenetically distant from Mnn9p, Van1p, and Anp1p in yeasts (Fig. 3), suggesting that they originally had the same function but acquired different physiological roles over the course of evolution. Another such example is the relationship between the GT15 family α-(1→2)-mannosyltransferase CmsA/CmsB and AfMnt1 in A. fumigatus (15–17, 28, 32). The α-(1→2)-/α-(1→6)-mannan of FTGM, a mannosyl chain unique to filamentous fungi belonging to Pezizomycotina, is biosynthesized by homologs of enzymes involved in synthesizing N- and O-glycans. The GT62 family members are widely conserved in the subphyla Saccharomycotina, Taphrinomycotina, and Pezizomycotina in Ascomycota, whereas such homologs are not conserved in Basidiomycota. The function of AnpA homologs may have diversified when they diverged and evolved from a common fungal ancestor in the phylum Ascomycota into budding and fission yeasts and filamentous fungi.

In yeast, Anp1p acts a member of the M-Pol II complex along with Mnn9p, Mnn10p, Mnn11p, and Hoc1p (23). However, disruption of *mnn9* and *van1* did not affect the FTGM α-core-mannan structure (Fig. 4), and single disruptants of *mnn10*, *mnn11*, and *hoc1* homologs did not exhibit a significantly altered phenotype (Fig. 1). No significant changes in N-glycan length were observed in the Δ*anpA* strain (Fig. S5). In addition, our recent study found that α-(1→6)-mannobiose is almost absent in O-glycan structures of the A. fumigatus A1151 strain. These findings indicate that AnpA is the only α-(1→6)-mannosyltransferase responsible for synthesizing the FTGM α-core-mannan structure in A. fumigatus. Du et al. reported that Mnn9 was important for cell wall mannan synthesis, although it did not majorly affect N-glycan structure (33). The detailed functions of the *mnn9*, *van1*, *mnn10*, *mnn11*, *och1-2*, *och1-4* genes in A. fumigatus remain unclear and should be further studied. Recently, OchC (also known as Och1-3) was characterized as a α-glucosaminide α-(1→6)-mannosyltransferase responsible for synthesizing Af3c, a zwitterionic glycosphingolipid (25). It is very interesting that homologs of GT genes in filamentous fungi have evolved to be responsible for the biosynthesis of glycan structures unique to filamentous fungi.

**FIG 7:**
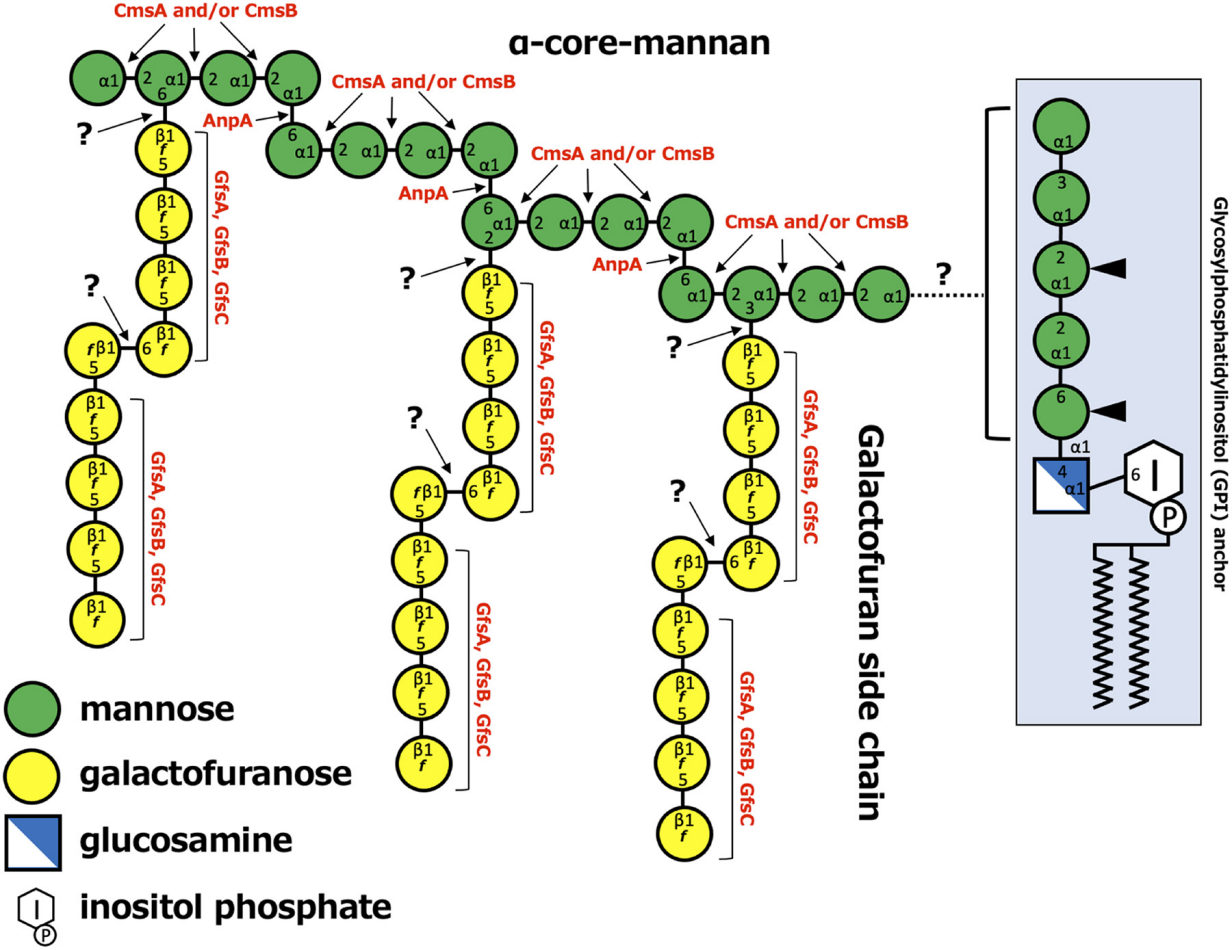
Summary model of fungal-type galactomannan (FTGM) biosynthesis in A. fumigatus. The structure of FTGM is depicted along with the enzymes attaching the mannose or galactofuranose residues. The enzymes at each step are represented in red text. CmsA and CmsB represent α-mannoside α-(1→2)-mannosyltransferase. GfsA, GfsB, and GfsC represent β-galactofuranoside β-(1→5)-galactofuranosyltransferases. AnpA represent α-mannoside α-(1→6)-mannosyltransferase. Question marks indicate where glycosyltransferases have not been identified.

Based on our results, we propose a model of FTGM biosynthesis in A. fumigatus (Fig. 7). FTGM appears to bind to any one of the mannose residues of the GPI anchor. The formation of α-(1→2)-mannosyl bonds with α-core-mannan is catalyzed by CmsA and/or CmsB (15). However, the relative contributions of CmsA and CmsB to the formation of the three of α-(1→2)-mannosyl linkages in each mannotetraose unit of the α-core-mannan structure remain unclear. The results of this study demonstrate that the transfer of α-(1→6)-mannosyl residues to α-core-mannan is catalyzed by AnpA. GfsA, GfsB, and GfsC are responsible for the biosynthesis of the galactofuran side chains of FTGM (5, 13, 14). Synthesized FTGMs on GPI anchors in the Golgi apparatus are transported by vesicles and localized to the cell surface (8–10). Transported FTGMs are thought to be digested and cross-linked with β-glucans by the DFG family of putative mannosidases (9, 10, 34), which have been hypothesized to hydrolyze the GPI core α-Man-(1→4)-d-glucosamine structure and catalyze the transfer of mannose oligosaccharides to β-glucans (34). Thus, the transfer of β-glucans to the mannan structure of FTGM may be important for hyphal growth in A. fumigatus; however, the mechanism by which FTGMs are transferred to GPI anchors has yet to be fully elucidated and warrants further study.

The disruption of *anpA* caused growth defects and the loss of the α-core-mannan structure in A. fumigatus (Fig. 1, Fig. S3, S4, and Table 1). Previously, multiple α-(1→2)-/α-(1→6)-mannosyltransferase mutant strains (including *anpA*) have been shown to be free of hyphal growth defects (26). This observation suggests that a compensation mechanism for cell wall integrity may be at work due to multiple mannosyltransferase gene disruptions. In fact, increased sensitivity of Δ*anpA* to CR and CFW was observed compared to the parental strain, suggesting that the disruption of *anpA* results in changes to the glucan and chitin content of the cell wall (Fig. 2C, panels b and c). This phenotype has previously been described after disruption of *cmsA*/*ktr4* (15, 16). In addition, the disruption of *cmsA*/*ktr4* and *cmsB*/*ktr7* increases the amount of unidentified glycosyl inositol phosphoceramides, thereby activating the synthesis of non-FTGM glycolipids by an unknown compensatory mechanism (9). Defects in FTGM also activate a compensatory mechanism for maintaining cell wall integrity.

In the mouse model of aspergillosis, disruption of *anpA* did not influence the virulence of A. fumigatus (Fig. S8), indicating that the complete structure of the α-core-mannan of FTGM is not essential for the pathogenicity of A. fumigatus in mice. In contrast, single gene disruption of *cmsA*/*ktr4* or *cmsB*/*ktr7* has been reported to reduce the pathogenicity of A. fumigatus in mice (16). The hyphae of Δ*anpA* have better growth than those of Δ*cmsA* and Δ*cmsB*. Thus, the loss of CmsA or CmsB may have a greater impact than the loss of AnpA on the pathogenicity of A. fumigatus in mice. However, the virulence of A. fumigatus is known to vary depending on the genetic background of the strains used and differences in the methods used to assess pathogenicity. Therefore, more detailed analyses of the contribution of FTGMs to the pathogenicity of A. fumigatus are required.

Three possible hypotheses may explain the low phenotypic impact of Δ*anpA* compared with the Δ*cmsA* and Δ*cmsB* phenotypes (Fig. 2C and Fig. S3) (15). Firstly, slight differences in the length of the remaining α-core-mannan structure of FTGM may account for the phenotypic differences between these strains. The short mannosyl chains synthesized by CmsA and CmsB may remain in the FTGM of the Δ*anpA* strain and contribute to maintaining cell wall integrity. Secondly, CmsA and CmsB may contribute to the synthesis of mannan structures (such as glycolipids) other than FTGM (9). The increased expression of CmsA and/or CmsB and increased synthesis of different mannan structures in the Δ*anpA* strain may help maintain the cell wall structure. Thirdly, a range of compensatory mechanisms may be present. Cell wall defects are known to be compensated for by enhanced biosynthesis of other glycans. Further, the compensatory mechanisms in Δ*anpA* may differ from those in the Δ*cmsA* and Δ*cmsB* strains. Accordingly, the biosynthesis of cell wall glycans remains a complex and poorly understood process that warrants further study.

Among the α-(1→6)-mannosyltransferases, only the crystal structure of yeast Mnn9p has been published (29). The structure includes bound Mn^2+^ and GDP, but does not provide information on the acceptor substrate (29). Recently, highly accurate protein structure prediction methods, such as AlphaFold2, have been developed (30). Van1p and Mnn9 from the budding yeast are required to form a heterodimer *in vivo* and *in vitro* (21, 22). On the contrary, AnpA was predicted to form a homodimer using AlphaFold2 (Fig. S6A and B), and the fact that recombinant AnpA was active *in vitro* on its own suggests that AnpA does not form heterodimers with other GTs; *in vivo*, it either functions alone or as a homodimer (Fig. 5A). The fact that the FTGM structure was not lost after disrupting *mnn9* or *van1* also supports this idea (Fig. 4). In this study, we also examined the structure–function relationship of AnpA using docking and MD simulations on the predicted structure. The highly accurate structure prediction by AlphaFold2 indicated that the overall structure of AnpA was similar to that of yeast Mnn9p, despite the amino acids in the N- and C-terminal regions being considerably different and that the amino acid residues of the Mn^2+^ ligand are highly conserved, but the surrounding residues, such as those forming the guanosine-binding pocket, are somewhat substituted (Fig. S2 and Fig. S6B and C).

Therefore, AnpA is considered to have diversified into fungal enzymes while retaining the structural basis for their α-(1→6)-mannosyltransferase activities. Docking and MD simulations further verified the predicted structure of AnpA as an α-(1→6)-mannosyltransferase and showed that the donor substrate GDP-Man and the acceptor substrate α-Man-OMe could be retained at the active site in the appropriate orientations (Fig. 6B and 6C). Although the reaction mechanism of α-(1→6)-mannosyltransferases has not been reported yet, the structural and quantum chemical studies of α-(1→2)-, α-(1→3)-, and α-(1→4)-GTs have suggested a front-side, substrate-assisted SNi-like reaction mechanism, likely common to the retaining GTs (35–37). The direct interaction and the orientation between GDP-Man and the 6-OH of α-Man-OMe observed in the AnpA simulations seems to be consistent with this catalytic mechanism. In the simulated structure of AnpA, the binding site of the terminal mannose of the acceptor substrate was located approximately 10 Å from the molecular surface (Fig. S7A), which seems to correspond to the α-core-mannan structure of FTGM, i.e., α-(1→2)-mannotetraose units linked by α-(1→6)-bonds. Figure S7B shows the superposition of the energy-minimized structure of α-Man-(1→2)-α-Man-(1→2)-α-Man-(1→2)-α-Man-OMe onto the α-Man-OMe of the complex by MD simulation. The superposition of the nonreducing terminal Man shows that the reducing terminal residue of α-Man-(1→2)-α-Man-(1→2)-α-Man-OMe reaches the molecular surface of AnpA, indicating that AnpA is unlikely to act on shorter α-(1→2)-mannosides *in vivo*. Recognizing the acceptor substrates of AnpA probably contributes to specifying the structure of the core-mannan of FTGM.

In conclusion, we identified an α-(1→6)-mannosyltransferase responsible for the biosynthesis of the α-core-mannan of FTGM. Our findings are expected to enhance the understanding of fungal cell wall structures and inform the development of new drugs against fungal pathogens in medicine and agriculture.

## MATERIALS AND METHODS

### Strains and growth conditions.

The A. fumigatus strains used in this study are listed in Table S1. The A. fumigatus strains were grown on Aspergillus minimal medium (MM).

10.1128/msphere.00484-22.9 TABLE S1 Strains used in the present study. Download Table S1, PDF file, 0.05 MB. Copyright © 2022 Kadooka et al. 2022 Kadooka et al. https://creativecommons.org/licenses/by/4.0/ This content is distributed under the terms of the Creative Commons Attribution 4.0 International license.

### Construction of the pHSG396-A. nidulans orotidine-5′-phosphate decarboxylase gene (*AnpyrG*) plasmid.

*AnpyrG* was amplified by PCR using A. nidulans A4 genomic DNA as the template and the primer pair pHSG396-AnpyrG-IF-F and pHSG396-AnpyrG-IF-R (primer sequences are provided in Table S2). The amplified fragment was inserted into the BamHI site of pHSG396 using the In-Fusion HD Cloning Kit (TaKaRa) to yield pHSG396-AnpyrG.

10.1128/msphere.00484-22.10 TABLE S2 Primers used in the present study. Download Table S2, PDF file, 0.04 MB. Copyright © 2022 Kadooka et al. 2022 Kadooka et al. https://creativecommons.org/licenses/by/4.0/ This content is distributed under the terms of the Creative Commons Attribution 4.0 International license.

### Construction of disruption strains.

To disrupt *mnn9*, *van1*, and *anp1* in A. fumigatus A1160, *AnpyrG* was inserted. A gene replacement cassette encompassing the homologous sequence at the 5′ terminal of *mnn9*, *van1*, *anp1*, *och1-1*, *och1-2*, *och1-3*, *och1-4*, *mnn10*, or *mnn11* and the homologous sequence at the 3′ terminal of *mnn9*, *van1*, *anpA*, *och1-1*, *och1-2*, *och1-3*, *och1-4*, *mnn10*, or *mnn11* was amplified by fusion PCR using A. fumigatus A1151 genomic DNA as the template and the primer pairs xxxX-1/xxxX-2 and xxxX-5/xxxX-6, respectively (Table S2). The *AnpyrG* marker was amplified by fusion PCR using pHSG396-AnpyrG as the template and the primer pair pHSG396-F/pHSG396-R. The DNA fragment amplified using the primers xxxX-1 and xxxX-4 was used to transform A. fumigatus A1160, yielding the Δ*mnn9*, Δ*van1*, Δ*anpA*, Δ*och1-1*, Δ*och1-2*, Δ*och1-3*, Δ*och1-4*, Δ*mnn10*, and Δ*mnn11* strains. MM agar plates without uracil and uridine were used to select transformants. The introduction of *AnpyrG* into each gene locus was confirmed by PCR using the primer pairs xxxX-F and xxxX-R (Fig. S1 and Table S2).

### Complementation of the *anpA* disruption strain carrying wild-type *anpA*.

For complementation analysis of *anpA* using a gene replacement cassette encompassing the homology arm at the 5′ terminal of *anpA* including wild-type *anpA*, the hygromycin B resistance gene (*hph*), and the homology arm at the *AnpyrG* were amplified by fusion PCR using A1151 genomic DNA, pHSG396-hygB, and pHSG-AnpyrG as templates and the primer pairs anpA-1/anpA-comp-1, pHSG396-F/pHSG396-R, and anpA-comp-2/anpA-comp-3, respectively. The DNA fragment, which was amplified using the primer pair anpA-1/anpA-comp-2 was used to substitute the Δ*anpA* locus with a complementary sequence, thereby yielding Δ*anpA*+*anpA*. MM agar plates containing 200 μg/mL hygromycin B were used to select transformants. The correct replacement of the DNA fragments for gene complementation was confirmed by PCR using the primer pair anpA-F/anpA-R (Fig. S4).

### Phylogenetic tree and multiple sequence alignment.

The phylogenetic tree was drawn in iTOL (38). Alignment and phylogenetic tree inference were performed with MAFFT 6.861 and RAxML 8.2.11 included in ETE v3.1.2 (39). Multiple sequence alignment was performed using ESPript (40) and Clustal W (41).

### Preparation of the FTGM fraction.

Total GM (FTGM and OMGM) from A. fumigatus was prepared according to a previously described method (5). A β-elimination reaction was performed according to a previously described method (5). To remove the galactofuran side chains, FTGMs were treated with 100 mM HCl at 100°C for 60 min.

### Proton ^1^H-NMR spectroscopy.

The ^1^H-NMR was performed according to a previously described method (5). Samples for ^1^H-NMR were exchanged twice in D_2_O with intervening lyophilization and then dissolved in D_2_O (99.97% ^2^H). ^1^H-NMR spectra were measured using a JNM-LA600 spectrometer (JEOL) at 45°C. Proton chemical shifts were referenced relative to internal acetone at δ 2.225.

### Construction of the pET15b-AnpA plasmid.

E. coli codon-optimized *anpA* (AFUB_063940) fragments from A. fumigatus A1163 were synthesized as gBlocks (Integrated DNA Technologies). The *anpA* fragments were cloned into the NdeI and NotI sites of pET15b-KAI (5) using the In-Fusion HD Cloning Kit (TaKaRa) to yield pET15b-AnpA, which was transformed into Shuffle T7 Express cells (New England Biolabs).

### Protein purification and quantification.

Bacterial expression and His-tagged protein purification were performed as described previously (5).

### Enzyme assays.

The artificial acceptor substrate *p*-nitrophenyl α-d-mannopyranoside (α-Man-pNP) was purchased from the Tokyo Chemical Industry Co., Ltd. Mannosyltransferase assays were performed as described previously (15). Standard assays were performed with α-Man-pNP (1.5 mM) as the acceptor, GDP-Man (5 mM) as the donor, and purified AnpA protein (0.1 μg/μL) in a total reaction volume of 40 μL. The mixture was incubated at 30°C for 16 h, and the reaction was stopped by heating at 99°C for 5 min. The supernatant was analyzed by reversed-phase high-performance liquid chromatography (HPLC) using an InertSustain C18 column (250 × 4.6 mm; GL Science). The elution was performed using 2 mobile phases as follows: solvent A, 5% acetonitrile in 20 mM triethylamine acetate (pH 7.0); and solvent B, 50% acetonitrile in 20 mM triethylamine acetate (pH 7.0). The gradient program was set at a flow rate of 0.8 mL/min (expressed as a percentage of solvent B) as follows: 0–10 min, 0%–40%; 10–30 min, 40%–90%; 30–35 min, 90%–0%; and 35–50 min, isocratic at 0%. Para-nitrophenol derivatives were detected by measuring the absorbance at 300 nm. Alpha-(1→6)-mannosidase was purchased from New England Biolabs and used according to the manufacturer’s instructions. To determine substrate specificity, α-mannobioses were used as substrate acceptors. Alpha-(1→2)-mannobiose and α-(1→6)-mannobiose were purchased from Dextra Laboratories Ltd. When mannobioses were used as acceptor substrates, the reaction products were analyzed using HPLC with a fluorescence detector after being labeled with ethyl 4-aminobenzoate (ABEE). Alpha-(1→2)-mannobiose or α-(1→6)-mannobiose derivatives were also labeled with ABEE, as previously described (42).

### Three-dimensional (3D) structure prediction of AnpA.

The 3D structure of the AnpA homodimer was predicted using AlphaFold version 2.2.0 with the AlphaFold-Multimer model weights (30, 43). AlphaFold version 2.2.0 was installed on a local Ubuntu computer using the procedure described on Github (https://github.com/deepmind/alphafold). By excluding the N-terminal region containing the transmembrane helix, the amino acid sequence from residue 43 to the C-terminus, which corresponds to the catalytic domain of AnpA, was submitted for prediction. Of the 25 predicted structures from the 5 AlphaFold-Multimer models, the structure with the highest confidence according to the predicted LDDT (pLDDT) score (43) was used as the final model for subsequent analyses. The quality of the final model was assessed using MolProbity (44) in addition to the pLDDT score.

### Docking and MD simulation of the AnpA complex with donor and acceptor substrates.

By superimposing the predicted apo dimer structure of AnpA on the previously reported structure of S. cerevisiae Mnn9p in complex with one GDP molecule and one Mn^2+^ ion at the active site (29), we manually modeled the active site of one monomer of AnpA with one Mn^2+^ ion. We performed a docking simulation between the AnpA dimer with Mn^2+^ ions and one GDP-Man molecule as a donor substrate using GNINA 1.0 (31) that is a fork of smina (45) and AutoDock Vina (46), a molecular docking program with integrated support for scoring and optimizing ligands using convolutional neural networks. Whole-protein docking using GININA 1.0 with the default parameters yielded 2 solutions with outstanding scores for the pose with GDP-Man bound to the active center of the dimer; thus, the structure with Mn^2+^/GDP-Man at the active site of 1 monomer was selected and used in the following analysis.

After energetic minimization of the Mn^2+^/GDP-Man complex model of the AnpA dimer using the GROMACS 2021.2 package (47), we performed a docking simulation between the complex and α-Man-OMe as its acceptor substrate using GNINA 1.0. The search space was defined as a box 15 × 15 × 15 Å in the x, y, and z directions centered between the Tyr231 and Asn232 side chains and encompassing the entire active site cavity of one monomer.

To evaluate the dynamic properties of the modeled structure of the enzyme–substrate complex, we performed MD simulations on the AnpA complex structure with the highest convolutional neural network pose score for the initial model. All simulations were performed using GROMACS 2021.2 with an Amber ff14SB force field (48) using previously reported topology and parameters for GDP-Man (49). The topology and parameter files for α-Man-OMe were generated using AmberTools17's LEaP (50) with the GLYCAM06-j force field (51). The system was explicitly solvated in a cubic box with TIP3P water models, periodic boundary conditions, and 24 Na^+^ ions added to neutralize the charge of the system. Amber topologies were created using AmberTools17’s LEaP and exported to GROMACS topologies by ACPYPE (52) before being subjected to energy minimization by the steepest descent. Following minimization, position-restrained MD simulations were performed, and the system was equilibrated in an NVT ensemble for 200 ps at 300 K using V-rescale temperature coupling, followed by an NPT ensemble for 200 ps at 1 atm using Berendsen pressure coupling. A 1.0-nm cutoff was applied for the short-range neighbor list, electrostatic, and van der Waals interactions. Long-range electrostatic interactions were measured using the Particle Mesh Ewald method. The equilibrated system was then subjected to a 500-ns production MD simulation using Parrinello–Rahman pressure coupling. Each simulation was performed three times. MD trajectories were analyzed using AmberTools17 and visualized using PyMOL (The PyMOL Molecular Graphics System, Version 2.0 Schrödinger, LLC) and VMD (53).

### Virulence assay.

The virulence assays in the mouse model (C57BL/6) were performed as previously described with a slight modification (16). Briefly, to suppress immunity, intraperitoneal administration of 150 mg/kg body weight cyclophosphamide (Shionogi & Company) and subcutaneous administration of 225 mg/kg body weight cortisone acetate (Wako Pure Chemical Industries) were performed 3 days and 1 day prior to infection, respectively. Subsequently, 150 mg/kg of cyclophosphamide was additionally injected on the second day of the infection. The body weight of each mouse was recorded weekly to adjust the dose of immunosuppressive drugs. Conidia of each strain were prepared by incubation on MM supplemented with 0.6 M KCl agar at 37°C for 5 days. On day 0, anesthesia was performed via subcutaneous injection (10 mL/kg) of a mixed solution containing 75 μg/mL of medetomidine hydrochloride (Domitor; Zenoaq Holdings Co.), 40 μg/mL of midazolam (Dormicum Injection 10 mg; Maruishi Pharmaceutical Co.), and 50 μg/mL of butorphanol tartrate (Butorphanol; Meiji Seika Pharma Co., Ltd.). While the anesthesia was in effect, 40 μL of phosphate-buffered saline containing 1.5 × 10^5^ conidia per mouse were inoculated intranasally. After the procedure, 75 μg/mL of atipamezole hydrochloride (Antisedan; Zenoaq Holdings Co.) was administered via intraperitoneal injection (10 mL/kg). The survival rate in mice was plotted against time. Differences between groups were compared by one-way analysis of variance.

### Data availability.

The materials and data generated in this study will be made available upon reasonable request to the corresponding author.
