# Supplementary figures and images for: Identification of an α-(1→6)-Mannosyltransferase Contributing To Biosynthesis of the Fungal-Type Galactomannan α-Core-Mannan Structure in Aspergillus fumigatus
Source: mSphere. 2022 Nov 29;7(6):e00484-22. doi: 10.1128/msphere.00484-22 (PMC9769593; doi:10.1128/msphere.00484-22)

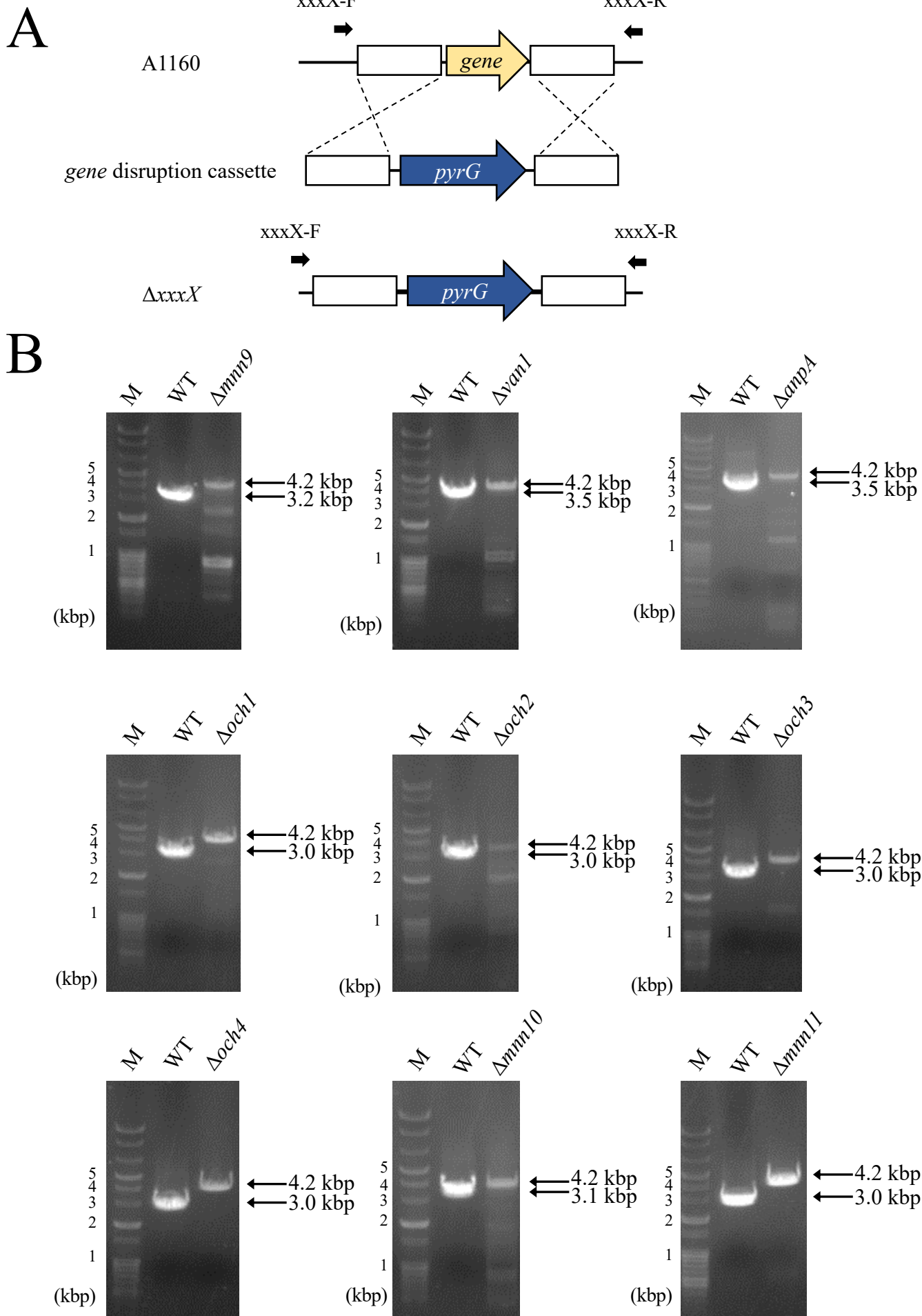

Kadooka et al. Fig. S1

Supplement: FIG S1 [file msphere.00484-22-s0001.pdf]

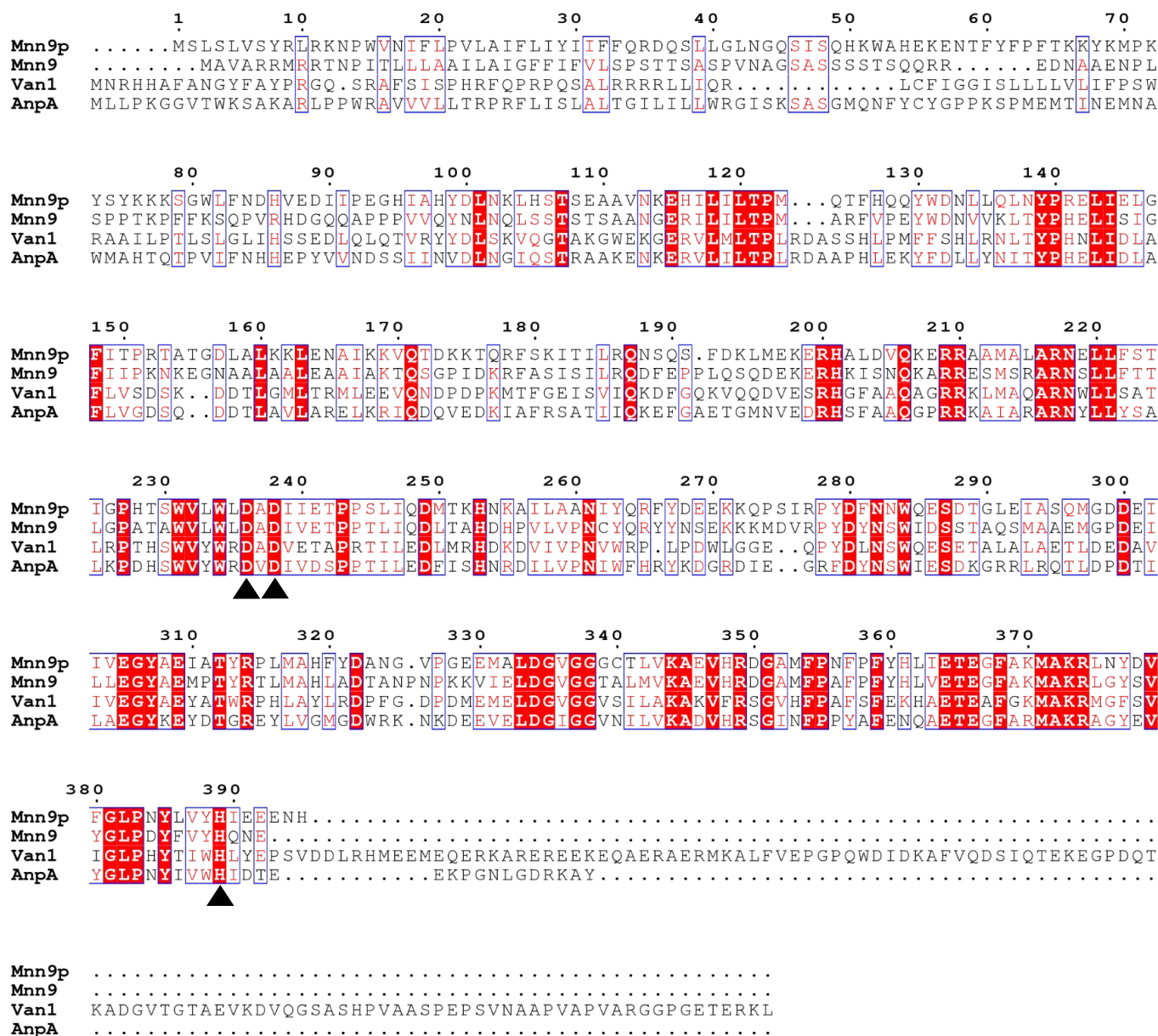

Supplement: FIG S2 [file msphere.00484-22-s0002.pdf]

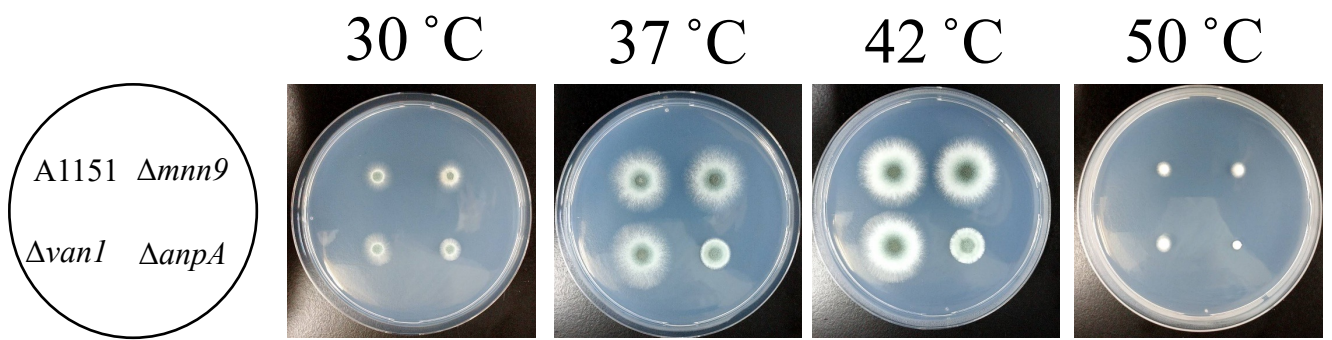

Supplement: FIG S3 [file msphere.00484-22-s0003.pdf]

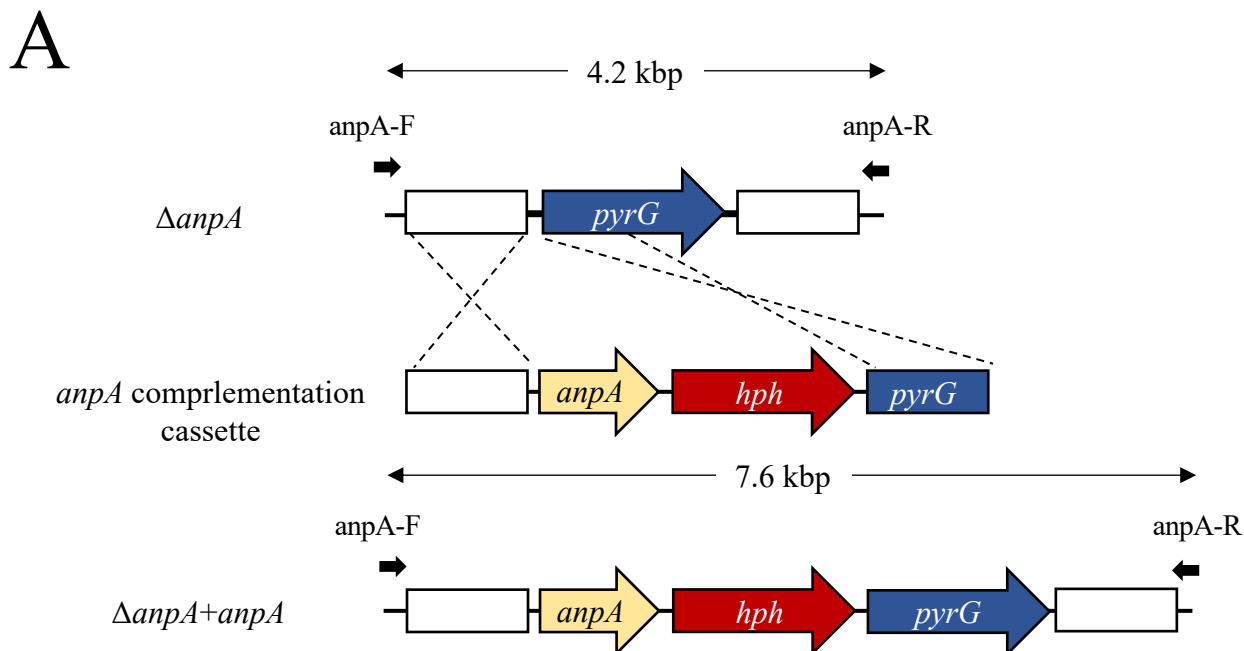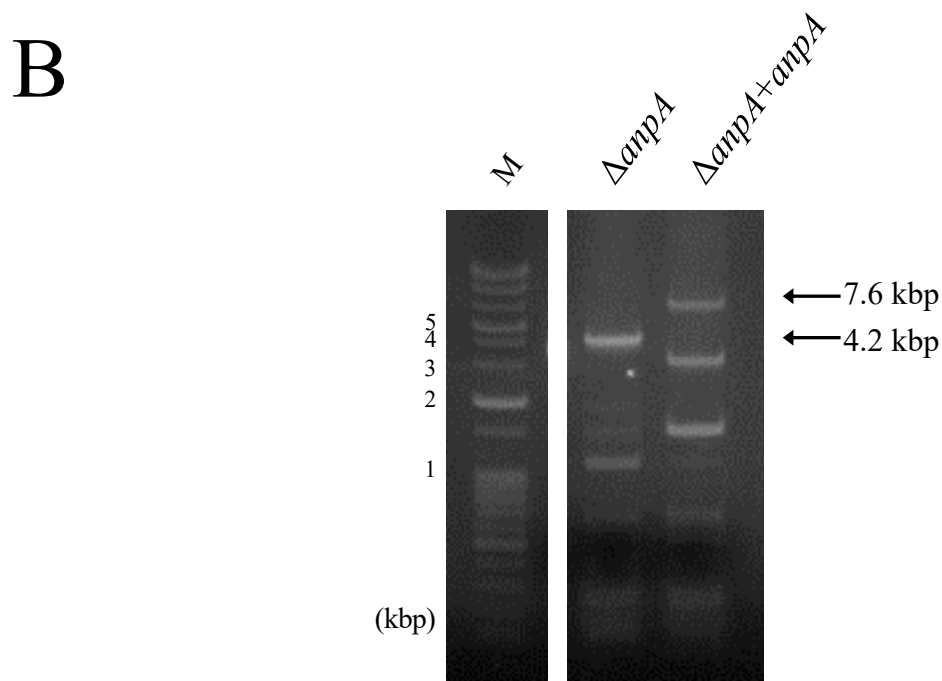

Supplement: FIG S4 [file msphere.00484-22-s0004.pdf]

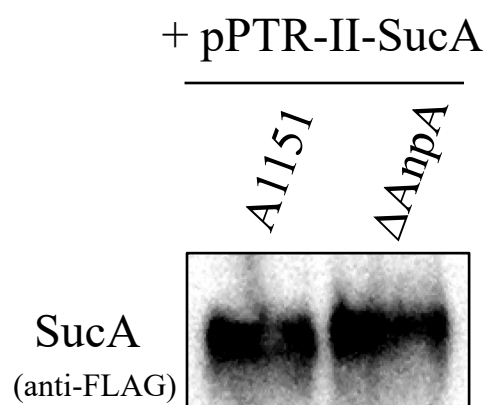

Supplement: FIG S5 [file msphere.00484-22-s0005.pdf]

A

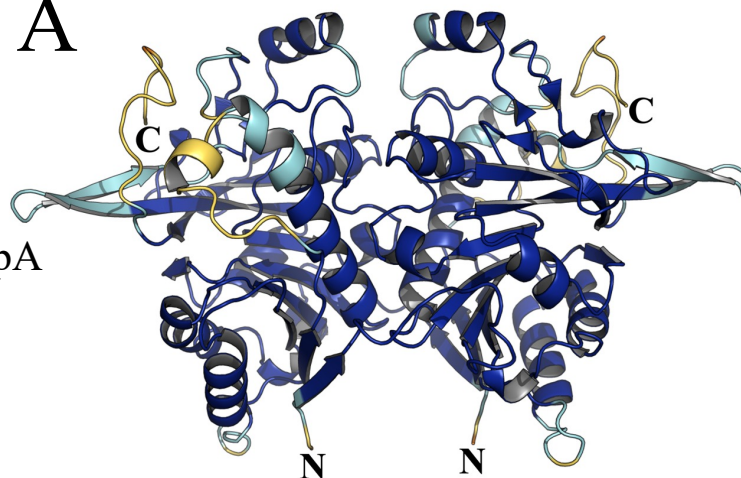*A. fumigatus* AnpA

B

*A. fumigatus* AnpA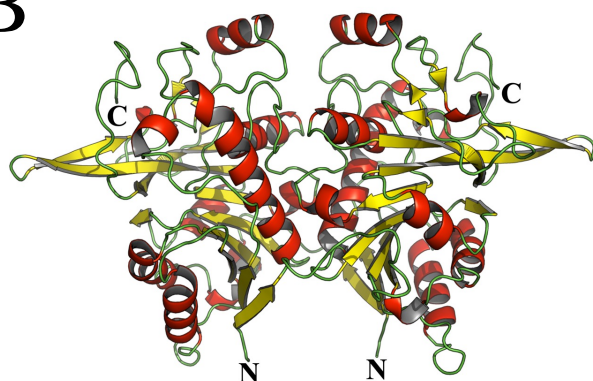

C

*S. cerevisiae* Mnn9p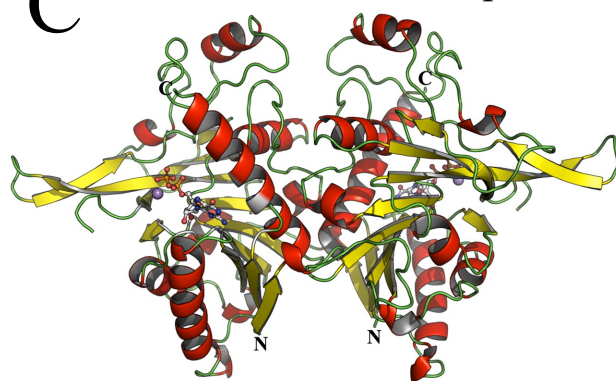

D

*S. cerevisiae* Mnn9p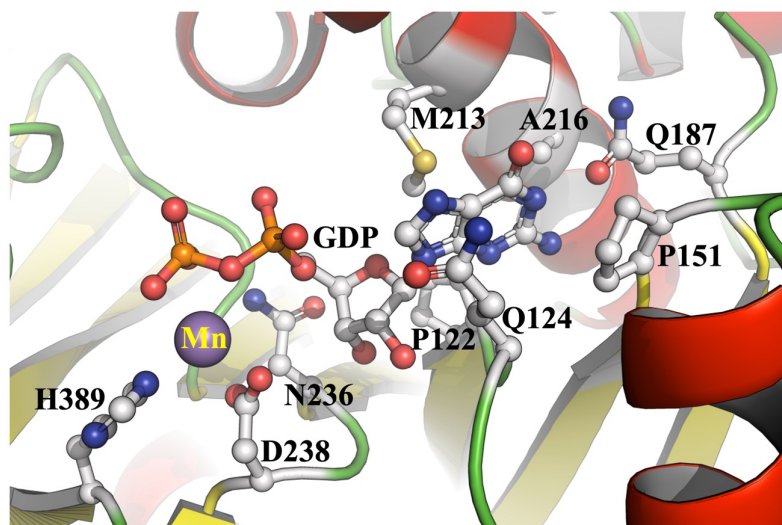

E

*A. fumigatus* AnpA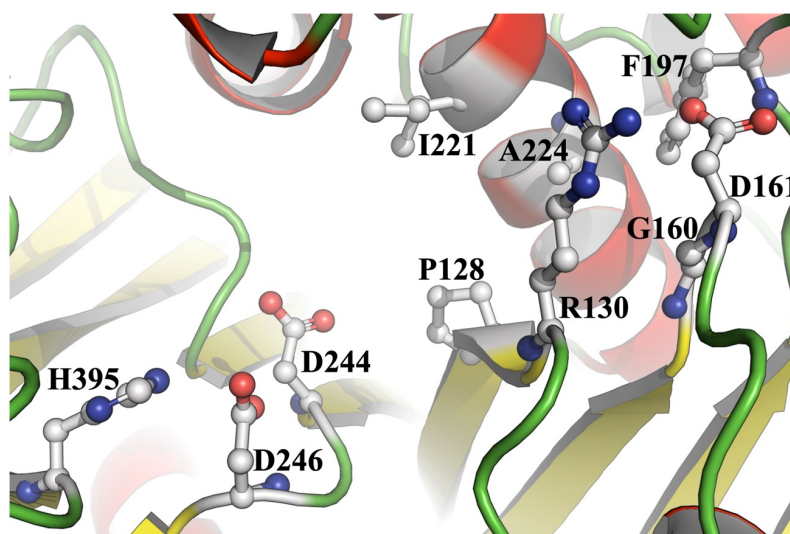

Supplement: FIG S6 [file msphere.00484-22-s0006.pdf]

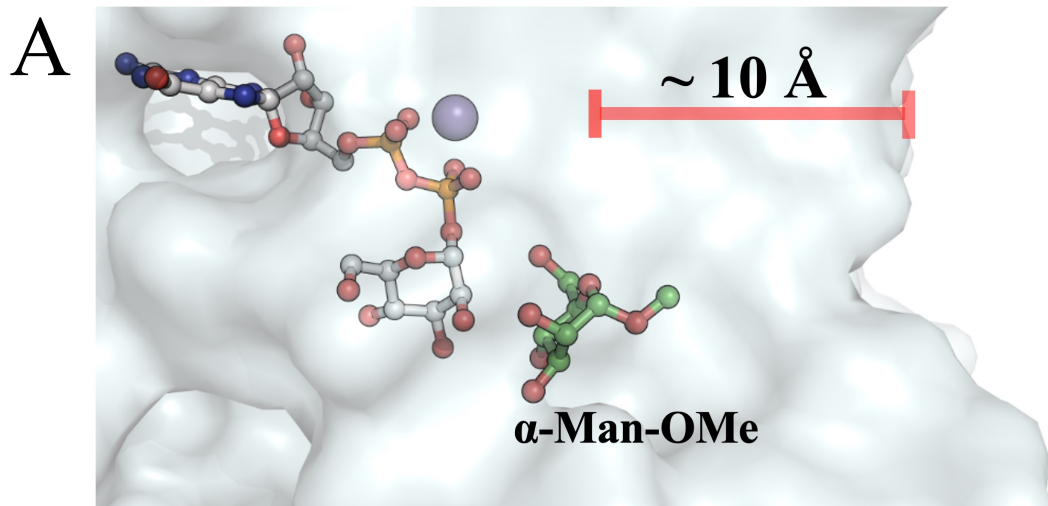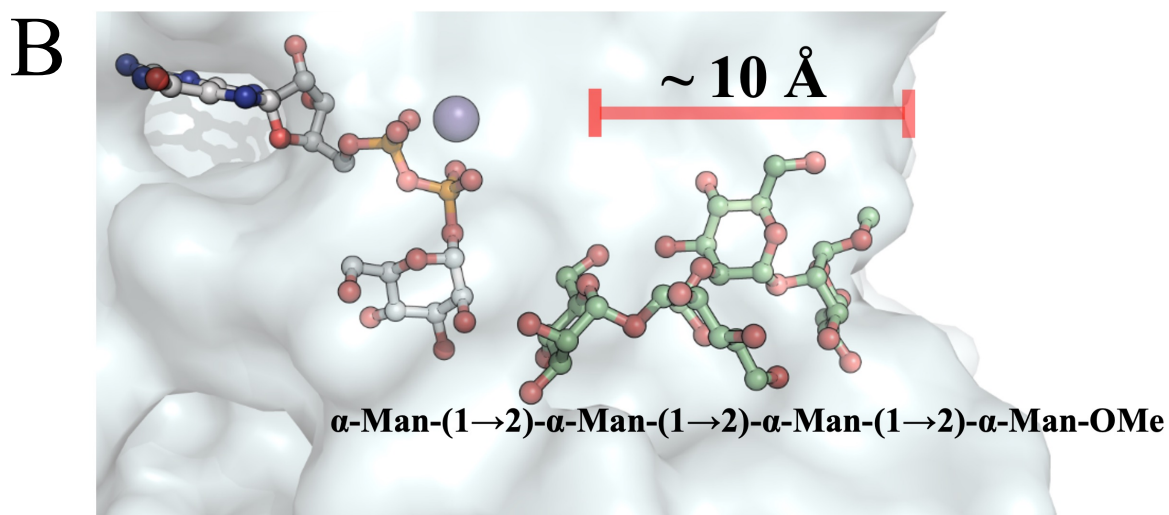

Fig. S7 Kadooka et al.

Supplement: FIG S7 [file msphere.00484-22-s0007.pdf]
