# Supplementary material for: Identification of an α-(1→6)-Mannosyltransferase Contributing To Biosynthesis of the Fungal-Type Galactomannan α-Core-Mannan Structure in Aspergillus fumigatus
Source: mSphere. 2022 Nov 29;7(6):e00484-22. doi: 10.1128/msphere.00484-22 (PMC9769593; doi:10.1128/msphere.00484-22)
Supplement: FIG S8 [file msphere.00484-22-s0008.pdf]

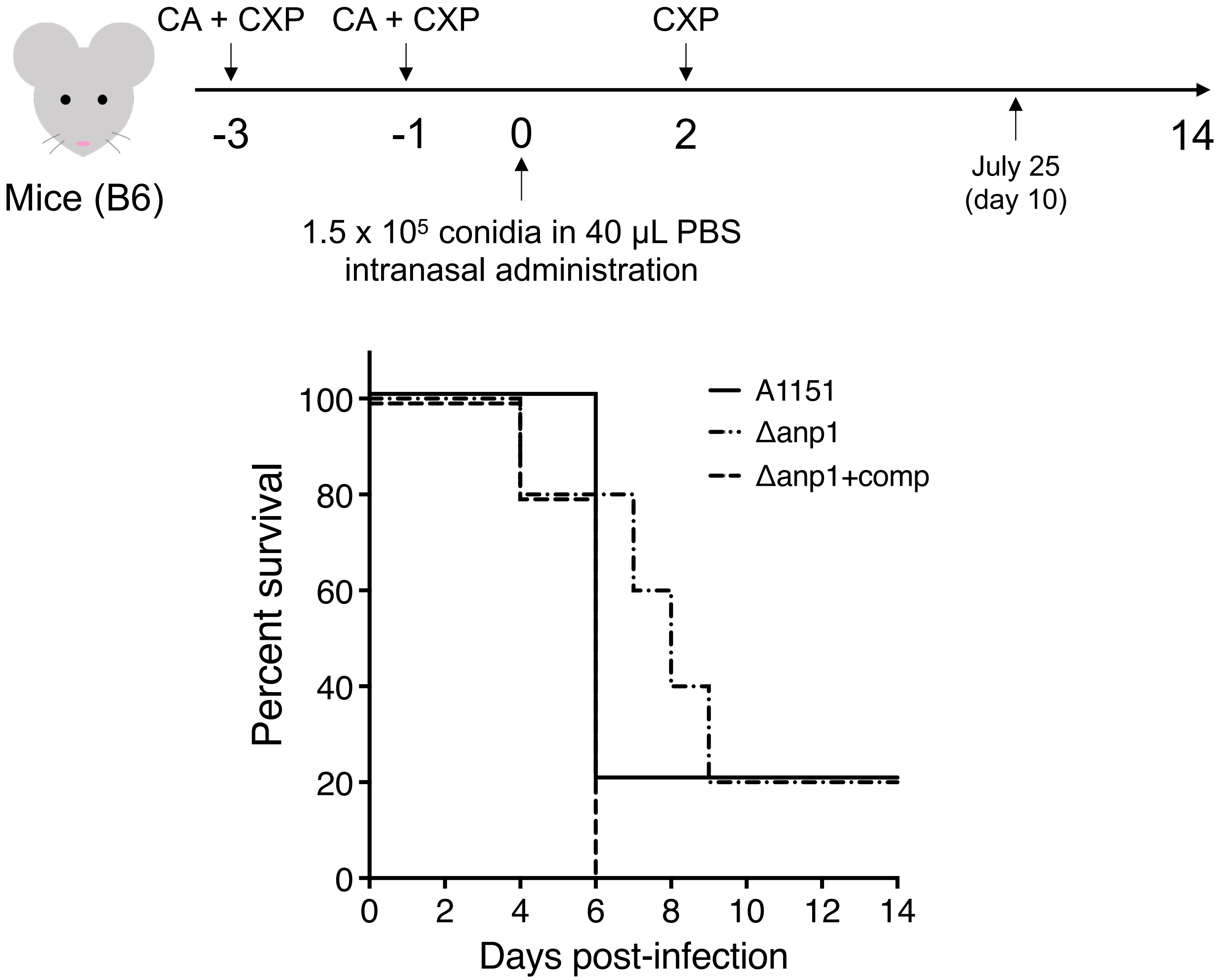

Cortisone acetate (CA), 225 mg/kg, subcutaneous administration
Cyclophosphamide (CXP), 150 mg/kg, intraperitoneal administration

Fig. S8 Kadooka et al.
