# Supplementary material for: Identification of an α-(1→6)-Mannosyltransferase Contributing To Biosynthesis of the Fungal-Type Galactomannan α-Core-Mannan Structure in Aspergillus fumigatus
Source: mSphere. 2022 Nov 29;7(6):e00484-22. doi: 10.1128/msphere.00484-22 (PMC9769593; doi:10.1128/msphere.00484-22)
Supplement: TABLE S1 [file msphere.00484-22-s0009.pdf]

Table S1. Strains used in the present study.

| Strains | Genotype | Source |
|---|---|---|
| A1151 | *pyrG* $^{AF}$::Delta KU80 | Obtained from FGSC |
| A1160 | DeltaKU80 *pyrG* - | Obtained from FGSC |
| Δ*mnn9* | DeltaKU80 *pyrG* - *mnn9* ::*AnpyrG* | This study |
| Δ*van1* | DeltaKU80 *pyrG* - *van1* ::*AnpyrG* | This study |
| Δ*anpA* | DeltaKU80 *pyrG* - *anpA* ::*AnpyrG* | This study |
| Δ*och1-1* | DeltaKU80 *pyrG* - *och1-1* ::*AnpyrG* | This study |
| Δ*och1-2* | DeltaKU80 *pyrG* - *och1-2* ::*AnpyrG* | This study |
| Δ*och1-3* | DeltaKU80 *pyrG* - *och1-3* ::*AnpyrG* | This study |
| Δ*och1-4* | DeltaKU80 *pyrG* - *och1-4* ::*AnpyrG* | This study |
| Δ*mnn10* | DeltaKU80 *pyrG* - *mnn10* ::*AnpyrG* | This study |
| Δ*mnn11* | DeltaKU80 *pyrG* - *mnn11* ::*AnpyrG* | This study |
| Δ*anpA* +*anpA* | DeltaKU80 *pyrG* - *anpA* ::*AnpyrG* ::*anpA* -*hph* -*AnpyrG* | This study |
| A1151+ *sucA* | A1151 harboring pPTR-II-SucA | Kadooka et al. 2022 |
| Δ*anpA* + *sucA* | Δ*anpA* harboring pPTR-II-SucA | This study |
