# Supplementary material for: Identification of an α-(1→6)-Mannosyltransferase Contributing To Biosynthesis of the Fungal-Type Galactomannan α-Core-Mannan Structure in Aspergillus fumigatus
Source: mSphere. 2022 Nov 29;7(6):e00484-22. doi: 10.1128/msphere.00484-22 (PMC9769593; doi:10.1128/msphere.00484-22)
Supplement: TABLE S2 [file msphere.00484-22-s0010.pdf]

Table S2. Primers used in the present study.

| Name | Sequence (5'-3') | Source |
|---|---|---|
| pHSG396-AnpyrG-IF-F | CGACTCTAGAGGATCCCCTTCAACGCTATCACGCC | This study |
| pHSG396-AnpyrG-IF-R | CATCGATGGGGGATCTCCCAGCCACAAAGGAACCT | This study |
| pHSG396-F | CGAGGGGTCGACTCTAGAGG | Kadooka et al. 2022 |
| pHSG396-R | TACCCCATCGATGGGGGATC | Kadooka et al. 2022 |
| mnn9-1 | CGGGCTGCAGGAATTGGCCGGGGGTATATCAGCT | This study |
| mnn9-2 | AGAGTCGACCCCTCGGACGACCGGTGAAAACGAGC | This study |
| mnn9-3 | CCCATCGATGGGGTAGCGGCTAGGGTACTCTGTTTA | This study |
| mnn9-4 | GCTTGATATCGAATTTCACCCACAACATCACCGAC | This study |
| mnn9-F | CGATGTCGGCGCCAGTAATT | This study |
| mnn9-R | GTCAAACTAGGCGGCTACCTC | This study |
| van1-1 | CGGGCTGCAGGAATTCTTGGACAGCGACTACGAGG | This study |
| van1-2 | AGAGTCGACCCCTCGCGGGGGTAGGCAAAGTAGC | This study |
| van1-3 | CCCATCGATGGGGTAGTGACAGGGACAGCAGAGGT | This study |
| van1-4 | GCTTGATATCGAATTGTGACGCGTTAGGCATTTCCT | This study |
| van1-F | GAAACAGTGGAGCTCTGGGG | This study |
| van1-R | GGTAGGTTCGGTTTGGAGTGC | This study |
| anpA-1 | CGGGCTGCAGGAATTCGCTCCGAACTGCATGCAAT | This study |
| anpA-2 | AGAGTCGACCCCTCGTATCAAGAATCGCGGCCGTG | This study |
| anpA-3 | CCCATCGATGGGGTAAACCAGGCTGAAACGGAAGG | This study |
| anpA-4 | GCTTGATATCGAATTACGCGATCTACTTCAATTGCCC | This study |
| anpA-F | TAAGCAGCTGTACACACCGG | This study |
| anpA-R | GGCAAAAGGACCGTGTCACA | This study |
| och1-1-1 | CGGGCTGCAGGAATTCGGTGGAGCCTGCAATCTTG | This study |
| och1-1-2 | AGAGTCGACCCCTCGGCGACTTCCTGAAGGTGAGC | This study |
| och1-1-3 | CCCATCGATGGGGTACCGGCTCTCTAAACGTCGAC | This study |
| och1-1-4 | GCTTGATATCGAATTTCTTCTCCGCATGTGTCACG | This study |
| och1-1-F | CAAGATCCAGAACGCTCCGT | This study |
| och1-1-R | CGCAGTGCTGCCTTCATTTC | This study |
| och1-2-1 | CGGGCTGCAGGAATTACATGTGAATTGGGTGGGCTC | This study |
| och1-2-2 | AGAGTCGACCCCTCGTGTGTGAATCGACGTGCCTTC | This study |
| och1-2-3 | CCCATCGATGGGGTACCGGCTTTGGCGGGTTTTAA | This study |
| och1-2-4 | GCTTGATATCGAATTTGAACTACTGTGCGCAGTGG | This study |
| och1-2-F | ACCCAAGTCTCACTGCCTGA | This study |
| och1-2-R | CAATGGGACAGACCATCCGTT | This study |
| och1-3-1 | CGGGCTGCAGGAATTATTTGCCAAGCTGCGAATGG | This study |
| och1-3-2 | AGAGTCGACCCCTCGGTAGTAGCGGCATCGTACGG | This study |
| och1-3-3 | CCCATCGATGGGGTATTTGGCCGGTGCAGAAATTG | This study |
| och1-3-4 | GCTTGATATCGAATTCCATCGTGGTGGATGTCGAC | This study |
| och1-3-F | CTGCTCGCTCATTGGCAGG | This study |
| och1-3-R | GCAGCGAATCCAAAGCAACA | This study |
| och1-4-1 | CGGGCTGCAGGAATTAGGGGTCATTGATCCCGACC | This study |
| och1-4-2 | AGAGTCGACCCCTCGGCGCTCCACAGATCCCAATC | This study |
| och1-4-3 | CCCATCGATGGGGTAGGAGCCTACCATGGCTGTGT | This study |
| och1-4-4 | GCTTGATATCGAATTAGCAGTCTGATACGCCTCCC | This study |
| och1-4-F | GACGAACGCACTATCAGGCA | This study |
| och1-4-R | CCGAGGGTTGTCACCATGAA | This study |
| mnn10-1 | CGGGCTGCAGGAATTGGCTCTGCGAACAATCTGGA | This study |
| mnn10-2 | AGAGTCGACCCCTCGGACGAGCTGACTTTGTCACC | This study |
| mnn10-3 | CCCATCGATGGGGTAAGAGTACATAAGCAGCGCCC | This study |
| mnn10-4 | GCTTGATATCGAATTTTCAGCAATGCCACTATGCC | This study |
| mnn10-F | GCCCTCGGAGAGTACCTCTT | This study |
| mnn10-R | CTACTGGTCTGCGGGCTTTTA | This study |
| mnn11-1 | CGGGCTGCAGGAATTATCCGTTAACACTGCAAGCTTC | This study |
| mnn11-2 | AGAGTCGACCCCTCGGGTGATCGAGAGAGGGACAT | This study |
| mnn11-3 | CCCATCGATGGGGTACAGCAGACTACTGCCAAAGG | This study |
| mnn11-4 | GCTTGATATCGAATTTCACCCATTAGCCATGCCAC | This study |
| mnn11-F | TTCAGGGTCCGATGCTTCTG | This study |
| mnn11-R | AGATAACCCTTGACCTTGCGC | This study |
| anpA-comp-1 | AGAGTCGACCCCTCGCTTTCAGGTCTACTTCGGC | This study |
| anpA-comp-2 | CCCATCGATGGGGTACCCTTCAACGCTATCACG | This study |
| anpA-comp-3 | GCTTGATATCGAATTGTAGTAACGTCTGCGGAGAC | This study |
| anpA-comp-R | CAGGATGTACGGTCAGCAAG | This study |
